# Synthesis of indano[60]fullerene thioketone and its application in organic solar cells

**DOI:** 10.3762/bjoc.20.109

**Published:** 2024-05-31

**Authors:** Yong-Chang Zhai, Shimon Oiwa, Shinobu Aoyagi, Shohei Ohno, Tsubasa Mikie, Jun-Zhuo Wang, Hirofumi Amada, Koki Yamanaka, Kazuhira Miwa, Naoyuki Imai, Takeshi Igarashi, Itaru Osaka, Yutaka Matsuo

**Affiliations:** 1 Department of Chemical Systems Engineering, Graduate School of Engineering, Nagoya University, Furo-cho, Chikusa-ku, Nagoya 464-8603, Japan; 2 Department of Information and Basic Science, Nagoya City University, Nagoya 467-8501, Japan; 3 Applied Chemistry Program, Graduate School of Advanced Science and Engineering, Hiroshima University 1-4-1 Kagamiyama, Higashi-Hiroshima, Hiroshima 739-8527, Japan; 4 Institute for Advanced Fusion, Resonac Corporation, 5-1 Okawa-cho, Kawasaki-ku, Kawasaki-shi, Kanagawa 210-0858, Japan; 5 Institute of Materials Innovation, Institutes for Future Society, Nagoya University, Furo-cho, Chikusa-ku, Nagoya 464-8603, Japan

**Keywords:** C_60_, evaporable fullerene derivatives, organic photovoltaics, organic solar cells, thioketone

## Abstract

Evaporable indano[60]fullerene ketone (FIDO) was converted to indano[60]fullerene thioketone (FIDS) in high yield by using Lawesson's reagent. Three compounds with different substituents in *para* position were successfully converted to the corresponding thioketones, showing that the reaction tolerates compounds with electron-donating and electron-withdrawing substituents. Computational studies with density functional theory revealed the unique vibrations of the thioketone group in FIDS. The molecular structure of FIDS was confirmed by single-crystal X-ray analysis. Bulk heterojunction organic solar cells using three evaporable fullerene derivatives (FIDO, FIDS, C_60_) as electron-acceptors were compared, and the open-circuit voltage with FIDS was 0.16 V higher than that with C_60_.

## Introduction

Fullerene is a carbon allotrope that has attracted significant scientific interest since its discovery by H. W. Kroto in 1985 [1]. Due to their distinctive spherical structure and electron-deficient properties, fullerene derivatives have found applications in various fields, including photovoltaics [2–5], biomedicine [6–8], and electron transporters [9–10]. Organic photovoltaic (OPV) and perovskite solar cell (PSC) technologies have proven to be promising candidates for the sustainable use of solar energy, with power conversion efficiency (PCE) improving to 17% for OPVs and over 25% for PSCs in just a few years [11]. Functionalized fullerene derivatives have played an important role both in OPVs as electron acceptors and in PSCs as electron transport layers (ETLs) by efficiently accepting electrons and hindering the transport of holes [12–14].

During the past 10 years, considerable attention has been focused on functional fullerene derivatives with an emphasis on tuning solubility and energy levels. Meanwhile, less attention has been devoted to improving the thermal stability of fullerene derivatives. One well-known example is [6,6]-phenyl-C_61_-butyric acid methyl ester (PCBM), which is recognized for its excellent solubility in solution-processed OPV fabrication [15]. Films generated through vacuum deposition, on the other hand, exhibit superior quality, have fewer defects, and are eco-friendlier than films produced by spin-casting. However, only a few studies to date have investigated the design of fullerene derivatives with the aim of improving their thermal stability, and more specifically, of designing evaporable fullerene derivatives [16–18]. Recently, we reported on perovskite solar cells fabricated with indano[60]fullerene ketone (FIDO), which was synthesized through fullerene cation chemistry. These cells demonstrated long-term stability and a remarkable PCE of 22.11%, surpassing that of the commonly used C_60_ (20.45%). This improved performance can be attributed to the evaporated amorphous film, which prevents the transformation of the film into a crystalline state during the heating and aging of the devices. Additionally, the ketone structure acts as a Lewis base, resulting in a passivation effect on Pb^2+^ [19].

In this study, we designed and synthesized indano[60]fullerene thioketones (FIDSs) with various *para*-substituents. The vacuum-deposition performance and thermal stability of FIDS were assessed by both normal-pressure and vacuum thermogravimetric analysis (TGA). Additionally, we conducted a comparative analysis of bulk heterojunction (BHJ) organic solar cells using the three evaporable fullerene derivatives investigated in this work.

## Results and Discussion

The synthesis of FIDO was performed by fullerene cation chemistry as reported by our group [20–26]. Conversion from ketone to thioketone is usually achieved by using Lawesson's reagent, which tends to form a trimer structure when reacted with indanone without a substituent at the α position [27–29]. Introduction of fullerene at the α position facilitated the successful transformation of ketone to thioketone.

Initially, we adopted the widely reported reaction conditions with 1.5 equiv of Lawesson's reagent and tetrahydrofuran (THF) as solvent. *t-*Bu-FIDO was dissolved in THF by sonication for 30 min. Unfortunately, the results were not fully satisfactory. Considering the poor solubility of fullerene derivatives, toluene, carbon disulfide (CS_2_), and *ortho*-dichlorobenzene (*o*-DCB) were tested as solvent. Surprisingly, the conversion from ketone to thioketone did not occur as anticipated. In another attempt, where the amount of Lawesson's reagent was increased to 3 equiv (Table 1, entry 5), the signal of thioketone was observed for the first time by high-performance liquid chromatography (HPLC) and MALDI time-of-flight mass spectrometry. Subsequently, a conversion of 99% was achieved by further increasing the amount of Lawesson's reagent and the reaction temperature. The transformation from ketone to thioketone was confirmed by ^13^C NMR, which showed a downfield shift from 198 ppm for the carbonyl carbon to 235 ppm for the thiocarbonyl carbon. With the optimized conditions (Table 1, entry 9) in hand, different *para*-substituents were used and the reaction from FIDO to FIDS was found to tolerate both electron-donating and -withdrawing functional groups as shown in Table 2.

**Table 1:** Optimization of reaction conditions for the treatment of *t-*Bu-FIDO with Lawesson's reagent.^a^

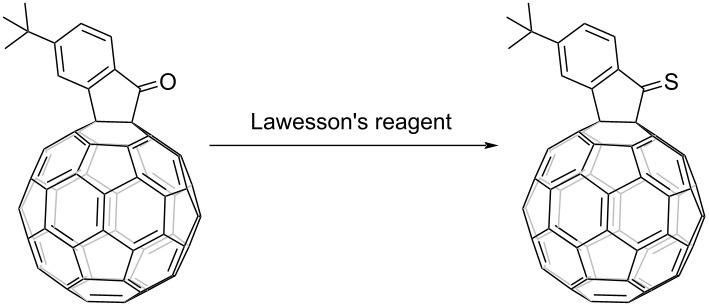					

Entry	Equiv	Reaction temperature (°C)	Solvent	Reaction time (h)	Conversion^b^ (%)

1 2 3 4 5 6 7 8 9 10	1.5 1.5 1.5 1.5 3 3 14 20 20 20	70 50 70 100 100 120 120 120 120 120	THF CS_2_ *o*-DCB toluene toluene toluene toluene toluene toluene *o*-DCB	14 14 14 14 14 14 14 14 20 20	NR^c^ NR NR NR 5 10 50 70 90 99

^a^All reactions were performed with *t-*Bu-FIDO (50 mg) under N_2_ atmosphere. All the solvents (10 mL) were anhydrous. ^b^Conversion was estimated from the HPLC peak area ratio. ^c^NR = no reaction.

**Table 2:** Reaction of R-FIDO with Lawesson's reagent.^a^

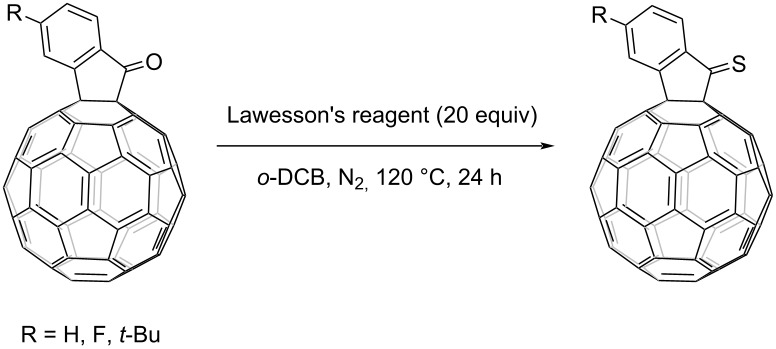				

Entry	R	Product	Yield^b^ (%)	Conversion (%)

1	*t-*Bu	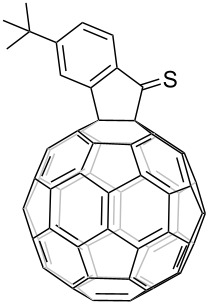	45	99
2	F	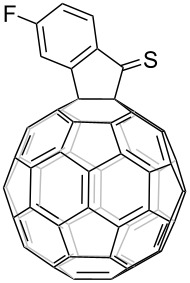	50	90
3	H	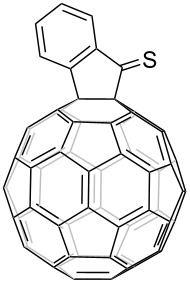	38	95

^a^All reactions were performed in *o-*DCB at 120 °C for 20 h under N_2_ atmosphere with FIDO as starting material. The molar ratio of Lawesson's reagent to FIDO was 20:1. ^b^Isolated yield.

**Figure 1:**
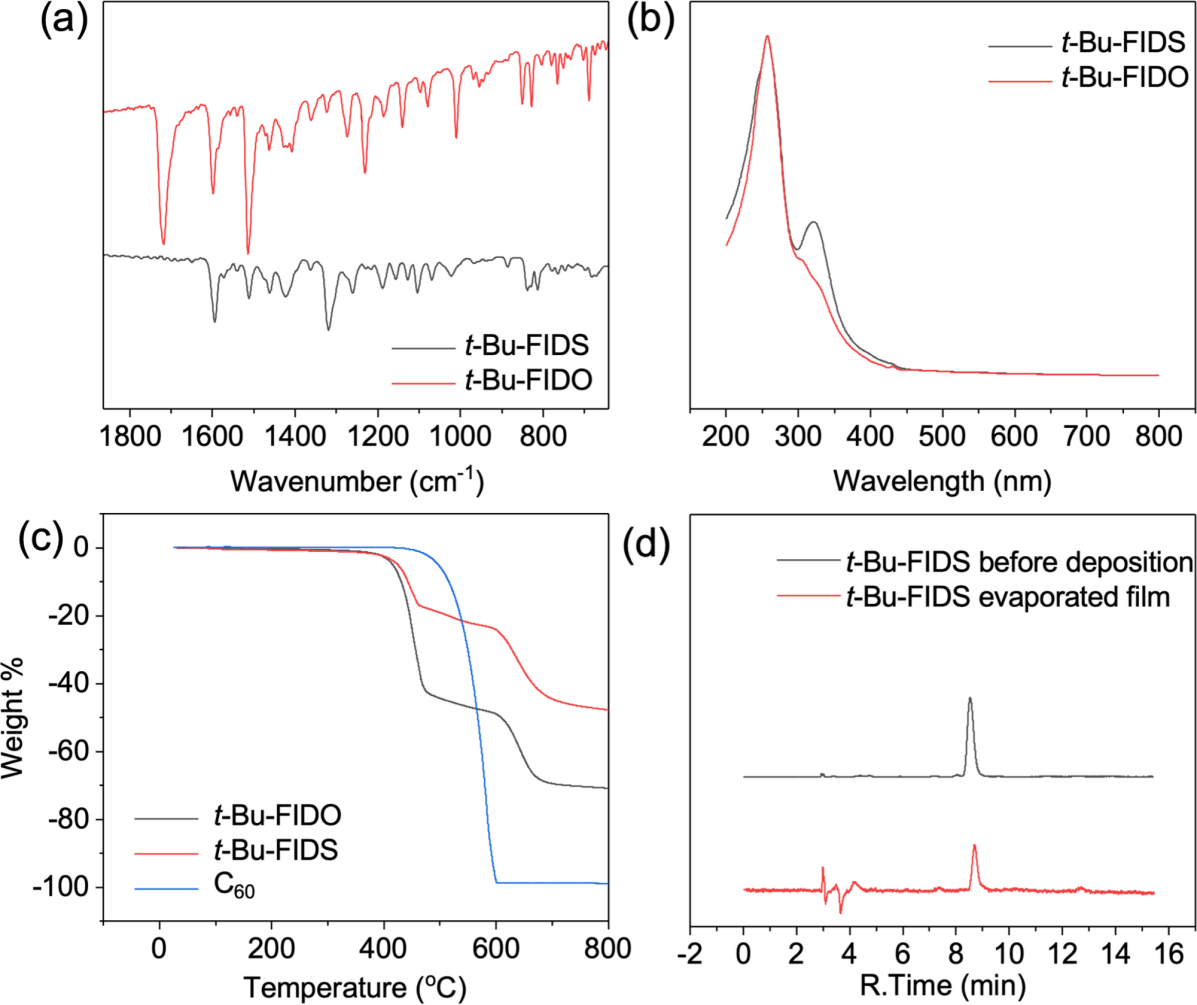
Characterization data. (a) FTIR spectra of *t*-Bu-FIDO and *t-*Bu-FIDS. (b) UV–vis spectra of fullerene derivatives normalized at 270 nm. (c) Vacuum TGA curves of *t*-Bu-FIDO (black), *t*-Bu-FIDS (red), and C_60_ (blue). The measurements were conducted under 0.1 Pa. (d) HPLC analyses before deposition of *t*-Bu-FIDS (black) and of toluene used to rinse the evaporated film of *t*-Bu-FIDS (red).

It is well known that functional groups with larger steric hindrance can reduce intermolecular forces. Consequently, a *tert-*butyl-functionalized compound (*t-*Bu-FIDS) was chosen for further studies in this work. To identify the formation of the thiocarbonyl group, Fourier transform infrared spectroscopy (FTIR) was conducted as shown in Figure 1a. The carbonyl stretching vibration peak of *t-*Bu-FIDO at 1720 cm^−1^ disappeared, indicating all the *t-*Bu-FIDO was completely consumed. Interestingly, the characteristic vibration peak of thiocarbonyl groups was not observed, which should be located at 1050–1300 cm^−1^ theoretically. Instead, numerous new low-intensity peaks were observed in this region.

To gain a comprehensive understanding of the differences between *t*-Bu-FIDO and *t-*Bu-FIDS, the density functional theory (DFT) method was employed using the B3LYP hybrid functional. The 6-31G* basis set was used for the geometry optimization and frequency calculation. The thiocarbonyl group in FIDS was found to have an out of plane bending vibration, however, the carbonyl group in FIDO showed a strong in-plane stretching vibration as shown in Figure S1 (Supporting Information File 1). Due to the direct connection between the five-membered ring and the fullerene cage, the out of plane bending vibration was easily affected by the fullerene cage vibration. Furthermore, the out-of-plane bending vibration of the thiocarbonyl group was also watched, which also affected the vibration of fullerene cage and benzene ring (Figure S2 and Table S1, Supporting Information File 1). This interesting phenomenon may explain the numerous new peaks that formed around 1000 cm^−1^ for *t*-Bu-FIDS.

Ultraviolet–visible (UV–vis) spectroscopy of *t-*Bu-FIDS in *o*-DCB exhibited two prominent UV absorption bands with peaks at 257 nm and 320 nm (Figure 1b). The absorption at 257 nm indicated the integrity of the fullerene cage chromophore. The absorption peak at 320 nm was assigned to the charge transfer band of the C=S bond in *t-*Bu-FIDS, which was stronger than that of the C=O bond in *t-*Bu-FIDO [30]. Interestingly, the maximum absorption band observed in *t-*Bu-FIDO at 432 nm, which is a characteristic feature of 58π-fullerene derivatives with a 1,2-addition pattern, was absent in *t*-*Bu*-FIDS [31]. Its absence might be due to perturbation caused by the presence of the C=S bond in *t*-Bu-FIDS.

To examine the sublimation behavior of *t-*Bu-FIDS under an environment similar to vacuum deposition, vacuum TGA measurements were performed using a TGA instrument connected to a vacuum pump and heater. The internal pressure of heater was reduced to 0.1 Pa or lower. Figure 1c shows the weight loss of the three fullerene derivatives under vacuum conditions. In contrast to C_60_, both *t*-Bu-FIDO and *t*-Bu-FIDS exhibited similar two-stage weight-loss curves. The sublimation temperature (*T*_sub_) was determined by analyzing the onset of weight loss for these compounds. *T*_sub_ was lower for *t-Bu*-FIDO and *t-Bu*-FIDS, at 410 °C and 417 °C, respectively, than for C_60,_ which began to sublimate at 460 °C (Table 3). The lower sublimation temperature was attributed to the steric hindrance of the *tert*-butyl groups, which disrupted the π–π stacking between the fullerene cages. The slight difference in sublimation temperature between *t*-Bu-FIDO and *t*-Bu-FIDS might be due to the slightly higher electron density of the sulfur atom compared with the oxygen atom. Additionally, data on the degradation temperature (*T*_deg_) obtained by normal-pressure TGA are shown in Table 3 and Figure S3 (Supporting Information File 1). *T*_deg_ was higher than *T*_sub_ for all three compounds, indicating their evaporability. Unfortunately, however, the sublimation window (*T*_deg_ –*T*_sub_) of *t*-Bu-FIDS was narrower than that of *t*-Bu-FIDO due to the lower *T*_deg_ of *t*-Bu-FIDS. A *t*-Bu-FIDS film was prepared by vacuum deposition at 0.1 Å/s, as shown in Figure 1d and Figure S4 (Supporting Information File 1). The film was uniform and smooth, with a thickness of approximately 20 nm. The film was rinsed with toluene, and the collected toluene was analyzed by HPLC to check for thermal decomposition during vacuum deposition. The *t*-Bu-FIDS structure remained almost unchanged, but very small amounts of two decomposed products were found at retention times of 7.5 min and 12.5 min. The latter decomposed product is C_60_, judged from the retention time.

**Table 3:** Sublimation and degradation temperatures of three evaporable fullerene derivatives.

Compound	*T*_sub_ (°C)	*T*_deg_ (°C)

C_60_ *t*-Bu-FIDO *t*-Bu-FIDS	460 410 417	– 474 450

**Figure 2:**
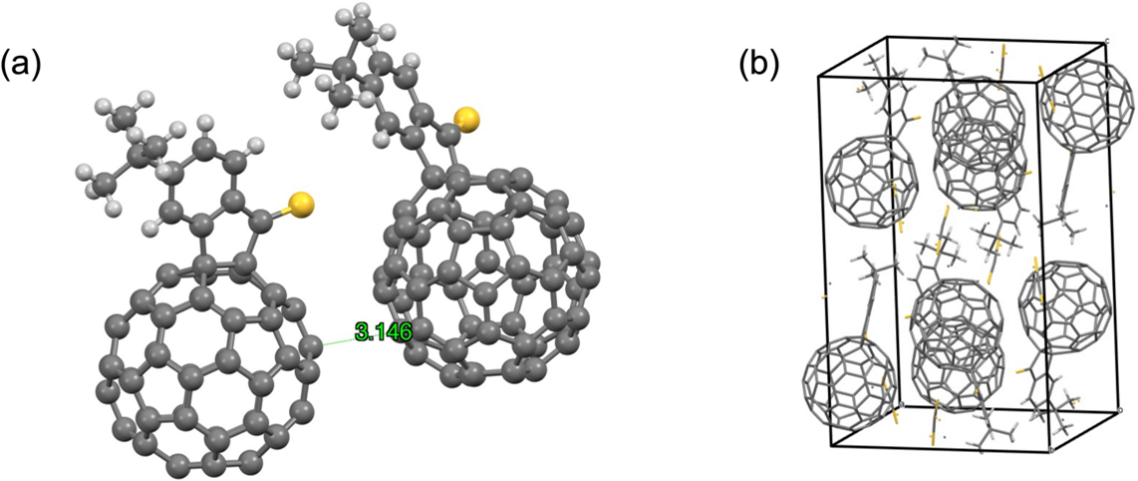
Single-crystal structure of *t*-Bu-FIDS. (a) The π–π distance between two molecules. (b) Crystal packing.

A single crystal of *t*-Bu-FIDS was grown by liquid–liquid diffusion method (CS_2_/EtOH). The crystal structure was successfully analyzed using synchrotron radiation at SPring-8. The crystal exhibited the orthorhombic space group (No. 61) with the *D*_2_*_h_* point group, and the structure confirmed the 1,2-addition pattern of *t*-Bu-FIDS (Figure 2a,b). The shortest π–π distance between the two fullerene molecules of *t*-Bu-FIDS in a unit cell was 3.14 Å, while that in *t*-Bu-FIDO was 2.974 Å [19]. The C=S bond length was 1.627 Å and was clearly longer than the C=O bond in *t*-Bu-FIDO. We consider that this longer bond length may have caused the higher reactivity and lower degradation temperature of *t*-Bu-FIDS compared with *t*-Bu-FIDO.

**Figure 3:**
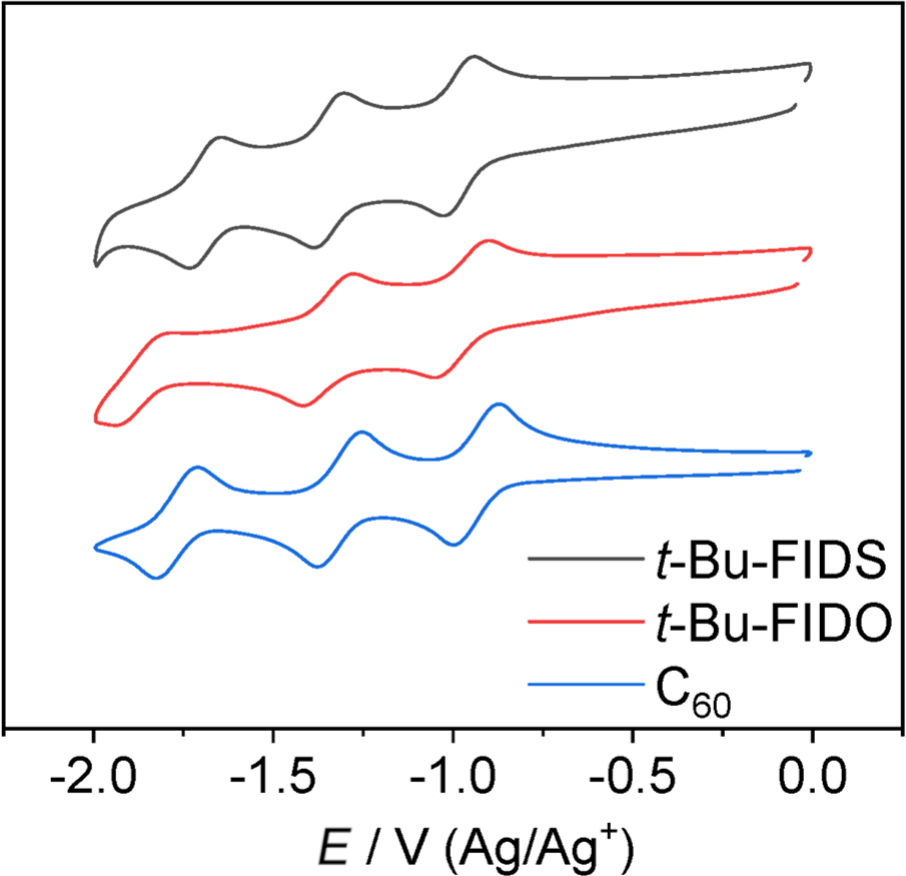
Cyclic voltammograms of fullerene derivatives in *o*-DCB solution containing Bu_4_N^+^(CF_3_SO_2_)_2_N^−^ (0.1 M) as supporting electrolyte at 25 °C with a scan rate of 0.05 V/s, for C_60_ (blue), *t*-Bu-FIDO (red), and *t*-Bu-FIDS (black). Glassy carbon, platinum wire, and Ag/Ag^+^ electrodes were used as the working, counter, and reference electrodes, respectively.

Electron-accepting ability is one of the most important properties for fullerene derivatives, and it is typically described in terms of the energy level of the lowest unoccupied molecular orbital (LUMO). To understand the electron affinity of *t*-Bu-FIDS, cyclic voltammetry was conducted. The cyclic voltammogram of *t*-Bu-FIDS in *o*-DCB showed reversible reduction waves at *E*_1/2_ = −1.14 V and −1.51 V (vs Fc/Fc^+^), as shown in Figure 3. Both the first and second reduction potentials of *t*-Bu-FIDS were higher than those of pristine C_60_. The LUMO energy of *t*-Bu-FIDS and C_60_ was calculated as −3.62 eV and −3.68 eV, respectively, using the following equation: *E*(LUMO) = −(*E*_1/2_^red1^ + 4.80) eV. To compare the electron affinity among the evaporable fullerenes, *t-Bu*-FIDO was also measured in the same solution system and showed a LUMO energy of −3.64 eV, which was slightly deeper than that of *t*-Bu-FIDS.

**Figure 4:**
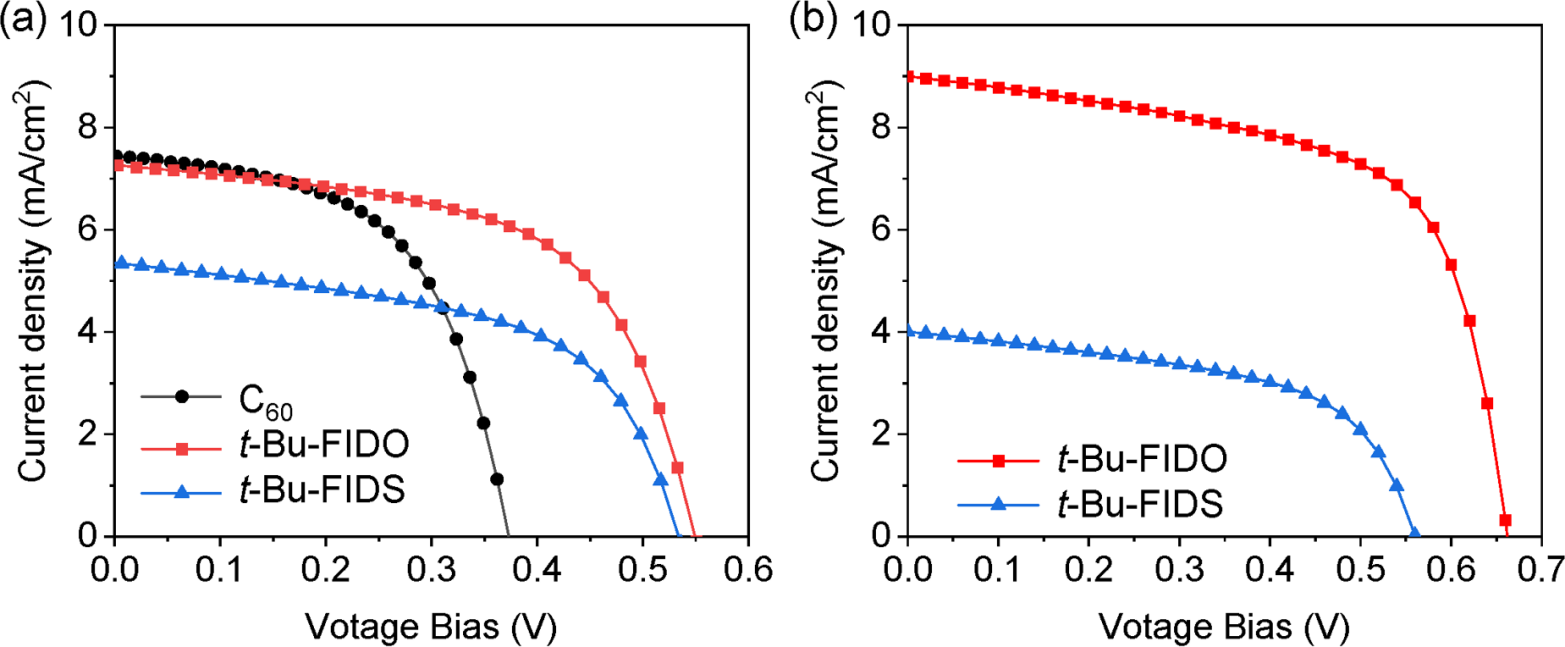
*J*–*V* curves of BHJ OPV devices. (a) ITO/ZnO/fullerene:P3HT (1:1, w/w)/PEDOT:PSS/Ag. (b) ITO/ZnO/fullerene:PNTz4T (2:1, w/w)/MoO_x_/Ag. ITO, indium tin oxide; PEDOT:PSS = poly(3,4-ethylenedioxythiophene) polystyrene sulfonate.

The use of vacuum-deposited FIDS as the electron transport layer in perovskite solar cells is still being explored. Considering that the open-circuit voltage (*V*_OC_) of OPVs is mainly determined by the difference between the HOMO level of the donor and the LUMO level of the acceptor, *t*-Bu-FIDS, with a higher LUMO level than C_60_, was used to fabricate a solution-processed BHJ OPV device with the donor poly(3-hexylthiophene) (P3HT). For comparison, the two known evaporable fullerenes C_60_ and *t*-Bu-FIDO were chosen. The results are summarized in Figure 4 and Table 4. Benefiting from higher *V*_OC_, the *t*-Bu-FIDS showed a PCE comparable to that of C_60_, although the short-circuit current density (*J*_SC_) was slightly lower. To further improve the performance of *t*-Bu-FIDO and *t*-Bu-FIDS, the crystalline polymer donor PNTz4T [32] was used. Compared with *t*-Bu-FIDS, *t*-Bu-FIDO achieved 3.71% PCE with larger *J*_SC_ and higher *V*_OC_. With PNTz4T, *t*-Bu-FIDS exhibited low performance, with *J*_SC_ of only 4 mA/cm^2^. This lower *V*_OC_ may be attributable to a suboptimal BHJ structure between PNTz4T and *t*-Bu-FIDS. Considering the lower *J*_SC_ performance of *t-*Bu-FIDS with both P3HT and PNTz4T, *t*-Bu-FIDS might not be suitable for BHJ OPV devices.

**Table 4:** Summary of photovoltaic parameters of BHJ OPV devices.

Fullerene	Donor	*V*_OC_ (V)	*J*_SC_ (mA/cm^2^)	FF	PCE %

C_60_ *t*-Bu-FIDO *t*-Bu-FIDS *t*-Bu-FIDO *t*-Bu-FIDS	P3HT*^a^* P3HT P3HT PNTz4T^b^ PNTz4T	0.37 0.55 0.53 0.66 0.56	7.45 7.53 5.39 9.00 4.00	0.55 0.60 0.55 0.62 0.54	1.55 2.47 1.58 3.71 1.23

^a^ITO/ZnO/fullerene P3HT (1:1, w/w)/PEDOT:PSS/Ag. ^b^ITO/ZnO/fullerene:PNTz4T (2:1, w/w)/MoO_x_/Ag.

## Conclusion

In summary, we successfully synthesized evaporable indano[60]fullerene thioketones with functional groups at the *para*-position of the benzene ring. Furthermore, we examined the sublimation behavior of three evaporable fullerene derivatives (FIDO, FIDS, C_60_). the sublimation window of *t*-Bu-FIDS was unfortunately slightly narrower than that of *t*-Bu-FIDO, which we have previously reported. Additionally, we compared the solution-processed OPV performance of the three evaporable fullerene derivatives as electron acceptors. FIDO exhibited the best performance, with *V*_OC_ that was 0.18 V higher compared with C_60_. Although FIDS showed a lower performance due to its less-desirable BHJ structure in the OPV, it could still potentially be utilized in perovskite solar cells in the future.

## Supporting Information

File 1 Experimental procedures, device fabrication, method of single-crystal growth, device evaluations and characterizations.

File 2 Chemical information file of *t-*Bu-FIDS, CCDC deposition number 2332797.

## Data Availability

The data that supports the findings of this study is available from the corresponding author upon reasonable request.
