# Supplementary material for: Synthesis of indano[60]fullerene thioketone and its application in organic solar cells
Source: Beilstein J Org Chem. 2024 May 31;20:1270–7. doi: 10.3762/bjoc.20.109 (PMC11181261; doi:10.3762/bjoc.20.109)
Supplement: File 1 — Experimental procedures, device fabrication, method of single-crystal growth, device evaluations and characterizations. [file Beilstein_J_Org_Chem-20-1270-s001.pdf]

BEILSTEIN JOURNAL OF ORGANIC CHEMISTRY

# Supporting Information

for

# Synthesis of indano[60]fullerene thioketone and its application in organic solar cells

Yong-Chang Zhai, Shimon Oiwa, Shinobu Aoyagi, Shohei Ohno, Tsubasa Mikie, Jun-Zhuo Wang, Hirofumi Amada, Koki Yamanaka, Kazuhira Miwa, Naoyuki Imai, Takeshi Igarashi, Itaru Osaka and Yutaka Matsuo

*Beilstein J. Org. Chem.* **2024,** *20,* 1270–1277. doi:10.3762/bjoc.20.109

# Experimental procedures, device fabrication, method of single-crystal growth, device evaluations and characterizations

License and Terms: This is a supporting information file under the terms of the Creative Commons Attribution License (https://creativecommons.org/licenses/by/4.0). Please note that the reuse, redistribution and reproduction in particular requires that the author(s) and source are credited and that individual graphics may be subject to special legal provisions.
The license is subject to the *Beilstein Journal of Organic Chemistry* terms and conditions: (https://www.beilstein-journals.org/bjoc/terms)

# Table of Contents

1. General S2

2. Synthesis of FIDSs S4

3. Simulation of IR spectra by Gaussian 16 S6

4. Thermal property characterized by TG-DTA S8

5. Crystal growth and single-crystal structural analysis S9

6. Photographs of films S10

7. NMR and MS spectra S11

8. Computational data S20

# 1. General

## Methods

To ensure optimal reaction conditions, all reactions that were sensitive to air or moisture were conducted in a dehydrated reaction vessel, under a carefully controlled nitrogen atmosphere. The transfer of air or moisture sensitive liquids and solutions was carried out using a syringe or Teflon cannula, which enabled accurate and precise delivery of the reagents. Prior to use, solvents were screened for their water content using the Karl-Fischer titration method with MKC-210 (Kyoto Electronics Manufacturing Co., Ltd.), and solvents should contain less than 30 ppm of water. Analytical thin-layer chromatography (TLC) was performed using a glass plate coated with 0.25mm 230–400 mesh silica gel containing a fluorescent indicator, allowing for precise detection and analysis of the reaction components. To remove the organic solutions, a diaphragm pump through a rotary evaporator was employed for evacuation. Nuclear Magnetic Resonance (NMR) was taken by ECS 400 and ECA 600 (JEOL Co., Ltd.) at room temperature reported in parts per million (ppm). $^{1}H$ NMR spectra were internally referenced to tetramethylsilane (0.00 ppm) or $CDCl_3$ (7.260 ppm). $^{13}C$ NMR spectra were internally referenced to tetramethylsilane (0.0 ppm) or $CDCl_3$ (77.16 ppm). The data were presented as following order: chemical shift, multiplicity (s = singlet, d = doublet, t = triplet, hept = heptet, m = multiplet and/or multiplet resonances), coupling constant in hertz (Hz), and signal area integration in natural numbers, assignment (italic). Matrix assisted laser desorption/ionization time-of- flight mass spectrometry (MALDI-TOF MS) was taken by AXIMA Performance (SHIMADZU Co., Ltd.). Super Photon ring-8 GeV (Spring-8) was employed to analyse the structure of single crystal of *t*-Bu-FIDS.

## Materials

Unless otherwise noted, all materials including dry solvents were obtained from commercial suppliers (Sigma-Aldric, TCI, Wako) and used without further purification.

## ***Photovoltaic device fabrication and evaluation***

### BHJ OPV Device fabrication

The OPV structure was fabricate using ITO/ZnO/Fullerene:P3HT (1:1, w/w)/PEDOT:PSS/Ag (ITO/ZnO/Fullerene:PNTz4T(2:1,w/w)/$MoO_x$/Ag). ITO-coated glass (sheet resistance ≤ 10 Ω $cm^2$) was cleaned consecutively in detergent, deionized water, acetone, isopropanol, and ethanol ultrasonic baths for 15 min, respectively. The ITO glass was treated with UV-Ozone for 15 min before use. BHJ solution was simply mixed P3HT and fullerene together in *o*-DCB at concentration 15 mg /ml (1:1 w/w) and keeping string 1 h at 60 $^oC$ before using (2:1 for fullerene: PNTz4T ). The 0.5% ZnO was prepared by dilution of 40% ZnO with ethanol, then keeping ultrasound about 20 min before using. To prepare the ZnO film, 2 drops of 0.5% ZnO solution was spin-coated on the substrates at 3000 rpm for 30 s, then the film was annealed at 80 $^oC$ for 10 min. Blend layer was deposited by spin coating the prepared blend solutions on the top of the ZnO layer at 1000 rpm for 30 s (600 rpm for 20 s for PNTz4T device). To get the better performance of the film, the substrates then was keeping under room temperature for 20 min. HTL then was deposited on the active layer with condition 3000 rpm for 180 s ($MoO_x$ was 7.5 nm by vacuum deposited for PNTz4T device). Finally, the Ag electrode (thickness about 100 nm) was deposited by thermal evaporation under high vacuum ($10^{-4}$ mbar). After fabrication, the device was annealed at 150 $^oC$ for 5 min.

### Evaluation of photovoltaic performance

The *J*–*V* measurement was performed via the solar simulator (HAL-C100, Asahi spectral) along with AM 1.5G spectra at 100 mW $cm^2$. The source unit (B2902A, Keysight) was used to measure the *J*–*V* curve.

# 2. Synthesis of FIDSs

## (1) *t*-Bu-FIDS

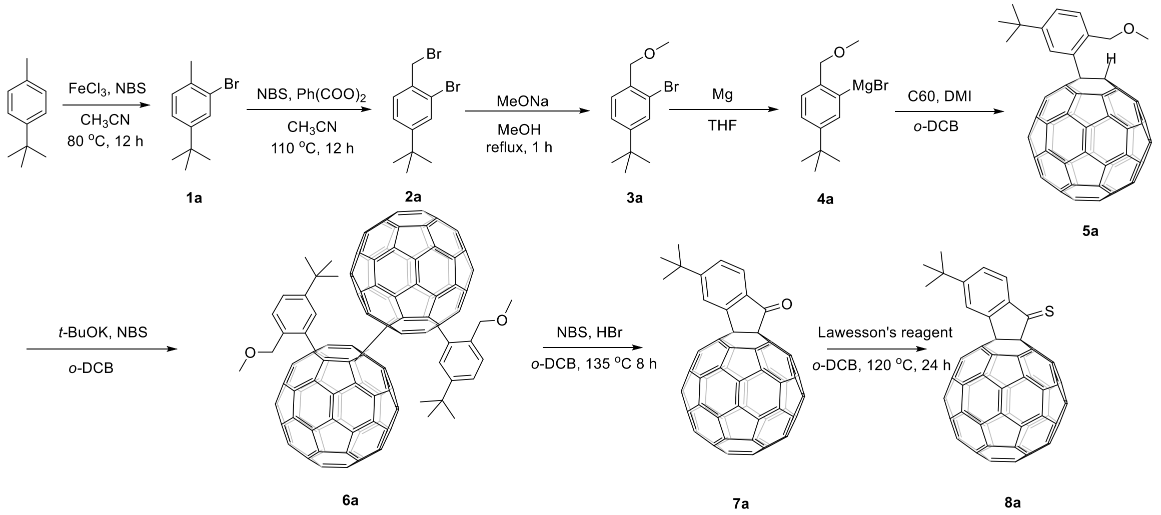


**Experiment details from 1a to 7a can be found in our previous work [1].**

**Synthesis of *t*-Bu-FIDS (8a)**. A mixture of **7a** (210 mg, 0.238 mmol) and Lawesson's reagent (1350 mg, 3.341 mmol) in 15 mL *o*-DCB (dry) was stirring at 120 °C for 24 hours under $N_2$ atmosphere. After cooled to room temperature, the reaction solution was poured into 300 mL of methanol to settle out the crude product. The filtered crude product then was purified by column chromatography on silica gel using $CS_2$ as eluent to afford ***t*-Bu-FIDS** as reddish brown solid. Yield: ***t*-Bu-FIDS**, 95 mg, 45%. MALDI-TOF MS *m/z* calcd for $C_{71}H_{12}S$ $[M]^+$ 896.06, found 897.05. $^{13}C$ NMR (101 MHz, CHLOROFORM-D) δ 233.47, 161.69, 156.41, 156.17, 152.88, 147.92, 147.66, 147.55, 146.56, 146.51, 146.30, 146.20, 146.05, 145.87, 145.61, 145.56, 145.52, 144.86, 144.49, 143.25, 142.97, 142.92, 142.71, 142.40, 142.21, 141.99, 141.92, 140.81, 140.31, 135.80, 135.01, 128.73, 128.14, 122.80, 36.29, 31.30. $^1H$ NMR (400 MHz, Chloroform-d) δ 8.47 (d, *J* = 8.4 Hz, 1H), 8.36 (d, *J* = 1.3 Hz, 1H), 7.83 (dd, *J* = 8.4, 2.1 Hz, 1H), 1.54 (s, 9H).

## (2) H-FIDS

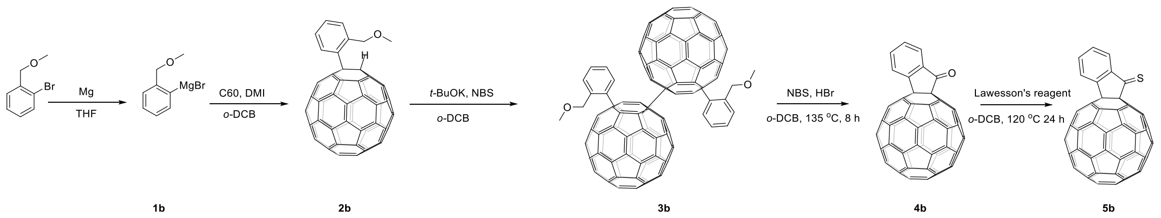


**Experiment details from 1b to 4b can be found in our previous work [1].**

**Synthesis of H-FIDS (5b).** A mixture of **4b** (130 mg, 0.157 mmol) and Lawesson's reagent (1276 mg, 3.159 mmol) in 15 mL *o*-DCB (dry) was stirring at 120 °C for 24 hours under $N_2$

atmosphere. After cooled to room temperature, the reaction solution was poured into 300 mL of methanol to settle out the crude product. The filtered crude product then was purified by column chromatography on silica gel using $CS_2$ as eluent to afford **H-FIDS** as reddish brown solid. Yield: **H-FIDS**, 50 mg, 38%. MALDI-TOF MS *m/z* calcd for $C_{67}H_4S$ $[M]^+$ 840.0, found 840.92. $^{13}C$ NMR (101 MHz, Chloroform-d) δ 234.56, 156.32, 155.89, 152.68, 147.83, 147.67, 147.57, 146.58, 146.32, 146.22, 146.03, 145.78, 145.64, 145.53, 144.85, 144.51, 144.33, 143.26, 142.99, 142.93, 142.72, 142.41, 142.38, 142.23, 142.02, 141.92, 140.80, 140.32, 136.65, 130.59, 128.36, 126.74. $^1H$ NMR (400 MHz, Chloroform-d) δ 8.55 (d, 1H, *J* = 7.9 Hz), 8.43 (d, 1H, *J* = 7.8 Hz), 8.06 (m, 1H), 7.80 (m, 1H).

**(3) F-FIDS**

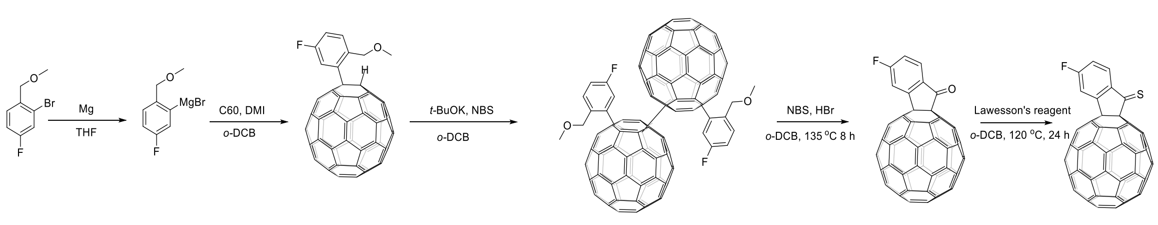


**Experiment details from 1c to 4c can be found in our previous work [2].**

**Synthesis of F-FIDS (5c).** A mixture of **4c** (70 mg, 0.083 mmol) and Lawesson's reagent (671.7 mg, 1.66 mmol) in 10 mL *o*-DCB (dry) was stirring at 120 °C for 24 hours under $N_2$ atmosphere. After cooled to room temperature, the reaction solution was poured into 300 mL of methanol to settle out the crude product. The filtered crude product then was purified by column chromatography on silica gel using $CS_2$ as eluent to afford **F-FIDS** as reddish brown solid. Yield: **F-FIDS**, 35.8 mg, 50% $^{13}C$ NMR (101 MHz, Chloroform-d) δ 232.46, 169.87, 167.25, 155.81, 151.93, 147.70, 147.66, 147.60, 146.61, 146.54, 146.33, 146.24, 145.98, 145.73, 145.62, 145.56, 145.53, 144.83, 144.47, 143.25, 143.00, 142.94, 142.70, 142.41, 142.23, 142.22, 142.02, 141.88, 140.87, 140.38, 135.95, 134.94, 130.74, 130.64, 119.38, 119.14, 113.60, 113.36, 89.40.$^1H$ NMR (400 MHz, Chloroform-d) δ 8.60-8.55 (m 1H), 8.11 – 8.04 (m, 1H), 7.52-7.48 (m, 1H). MALDI-TOF MS *m/z* calcd for $C_{67}H_3FS$ $[M]^+$ 857.99, found 857.66.

# 3. Simulation of IR spectra by Gaussian 16

Frequency calculation was using DFT B3LYP functional with a basis set of 6-31G*. [3-5] All the calculation was conducted by Gaussian 16. [6]

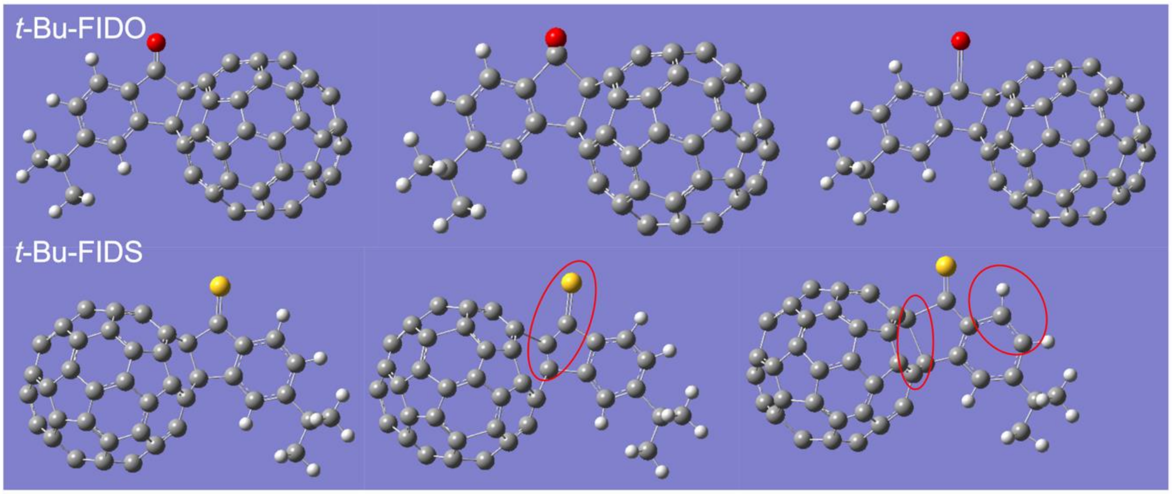


**Figure S1.** The simulated vibration *t*-Bu-FIDO and *t*-Bu-FIDS.

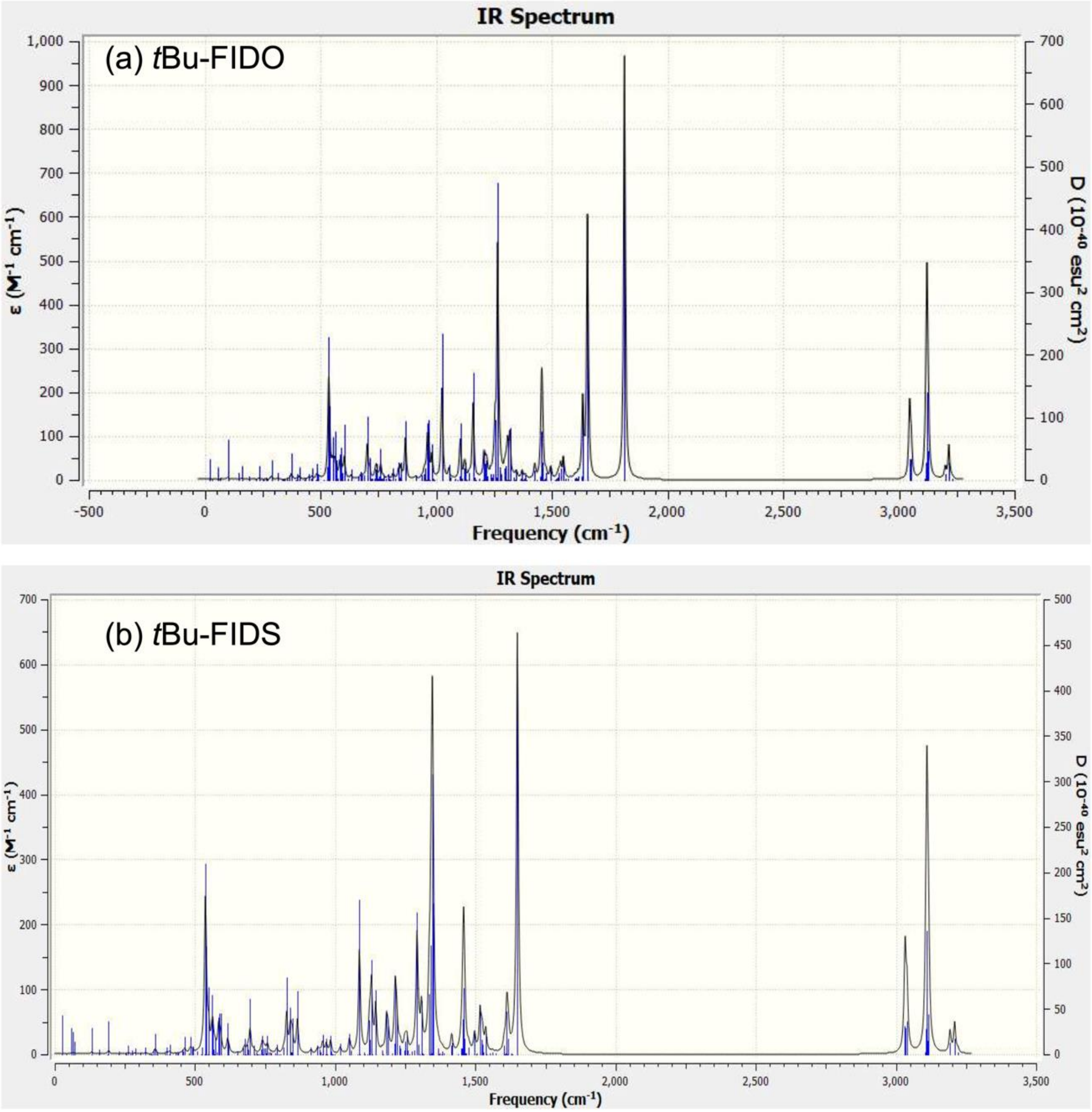


**Figure S2.** The simulated vibration frequency of (a) *t*-Bu-FIDO and (b) *t*-Bu-FIDS.

## 4. Thermal property characterized by TG-DTA

### *Method and procedure of TG-DTA:*

Thermogravimetry Thermal Analysis (TG-DTA) is taken by DTG-60AH (SHIMADZU CO.). It is worth mentioning that the sample used for TG-DTA characterization is fullerene powder after removing the organic solvent. Under a $N_2$ gas flow with temperature ramp rate of 10 $^oC$ /min from rt to 800 °C.

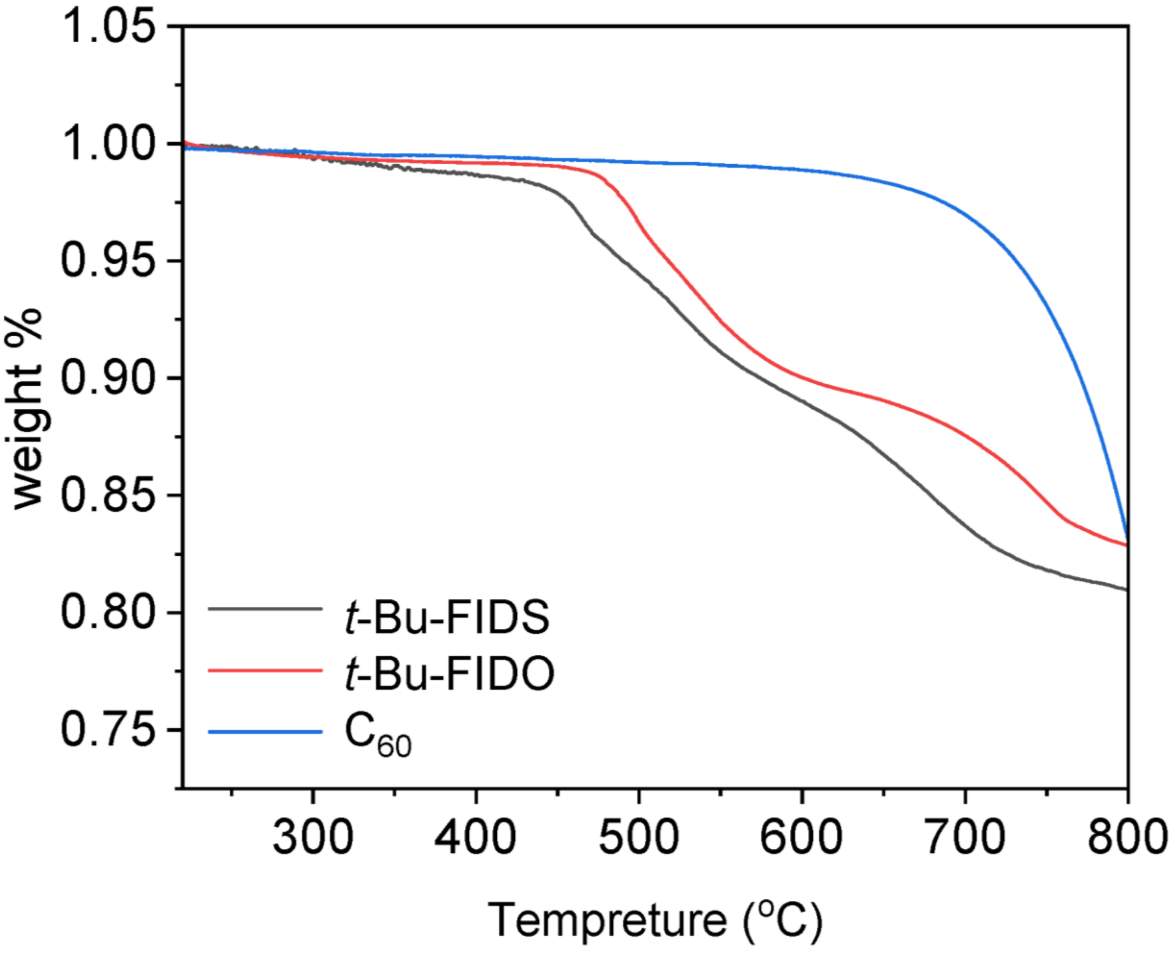


**Figure S3.** Normal pressure TGA of C60 (Bule), *t*-Bu-FIDO (Red) and *t*-Bu-FIDS (Black).

## 5. Crystal growth and single-crystal structural analysis

Single crystals of *t*-Bu-FIDS were successfully grown by liquid-liquid diffusion method. *t*-Bu-FIDS was dissolved in carbon disulfide at a concentration of 1 mg/mL and then transferred into the narrow receiver. An equal volume of ethanol was slowly added to a narrow receiver via a subcutaneous syringe, resulting in the formation of clearly distinct layers. The system was then sealed and left undisturbed for a period of 14 days. Single crystal X-ray diffraction data was collected at 100 K using the diffractometer with a DECTRIS EIGER X 16M detector at beamline BL41XU of SPring-8 large synchrotron radiation facility (Hyogo, Japan). X-ray structure analysis of *t*-Bu-FIDS: $C_{71}H_{12}S$, 100 K, orthorhombic, $P_{bca}$, $a$ = 17.4935(2) Å, $b$ = 16.1506(2) Å, $c$ = 28.4847(2) Å, $V$ = 8047.80(15) Å$^3$, $Z$ = 8, $R$ (w$R$) = 0.0747 (0.1748) for 727 parameters and 19499 unique reflections. Cambridge Crystallographic Data Center (CCDC) deposition number is 2332797.

## 6. Photographs of films

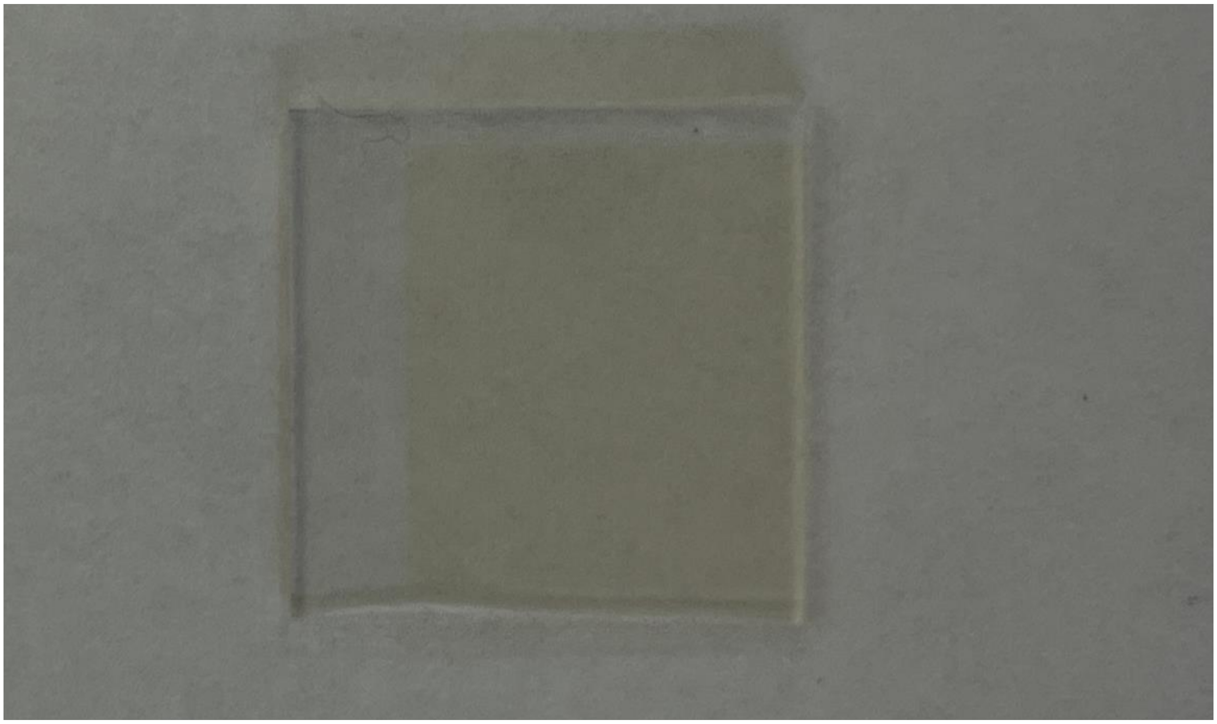

**Figure S4.** Photograph of evaporated *t*-Bu-FIDS film.

# 7. NMR and MS spectra

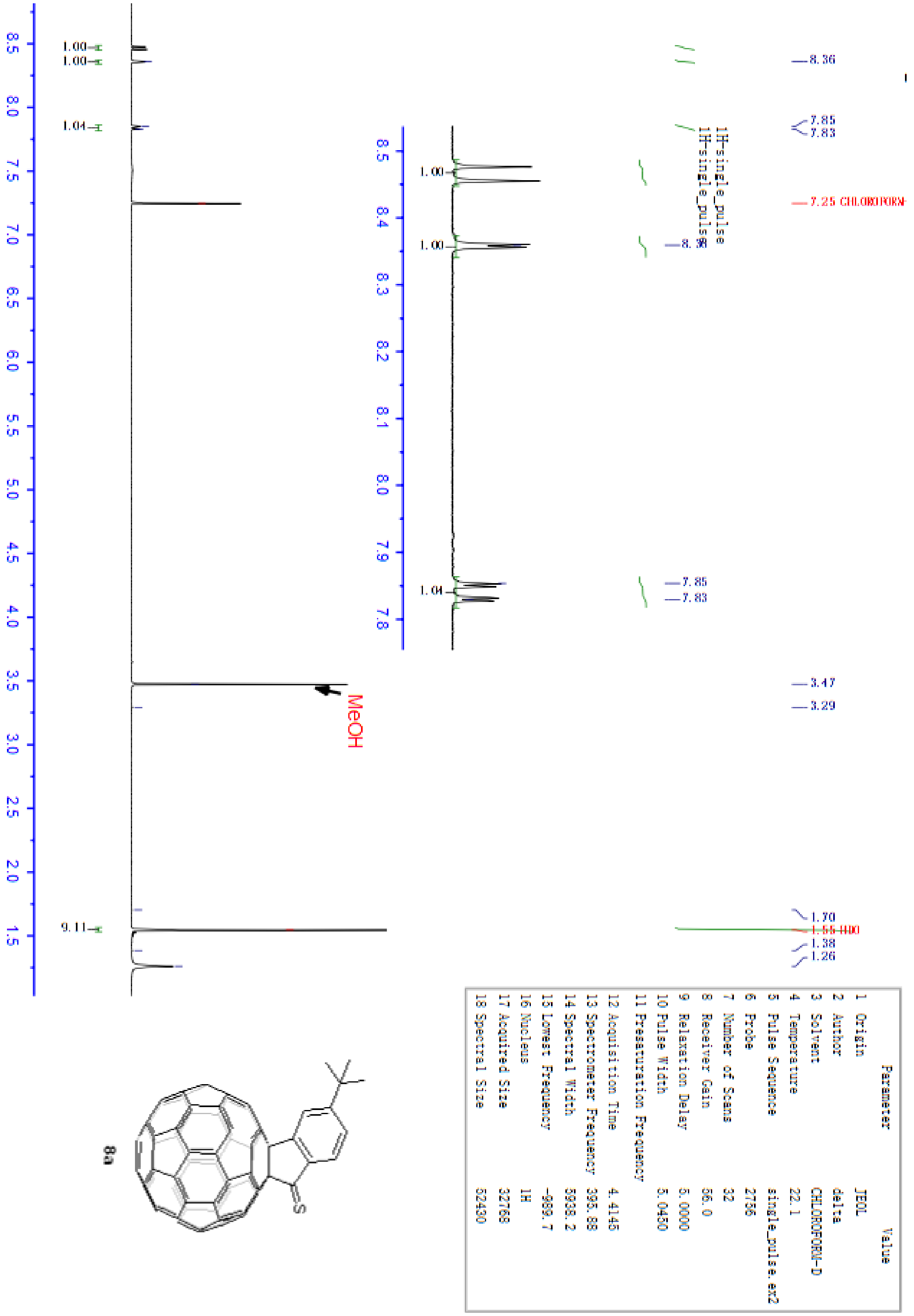

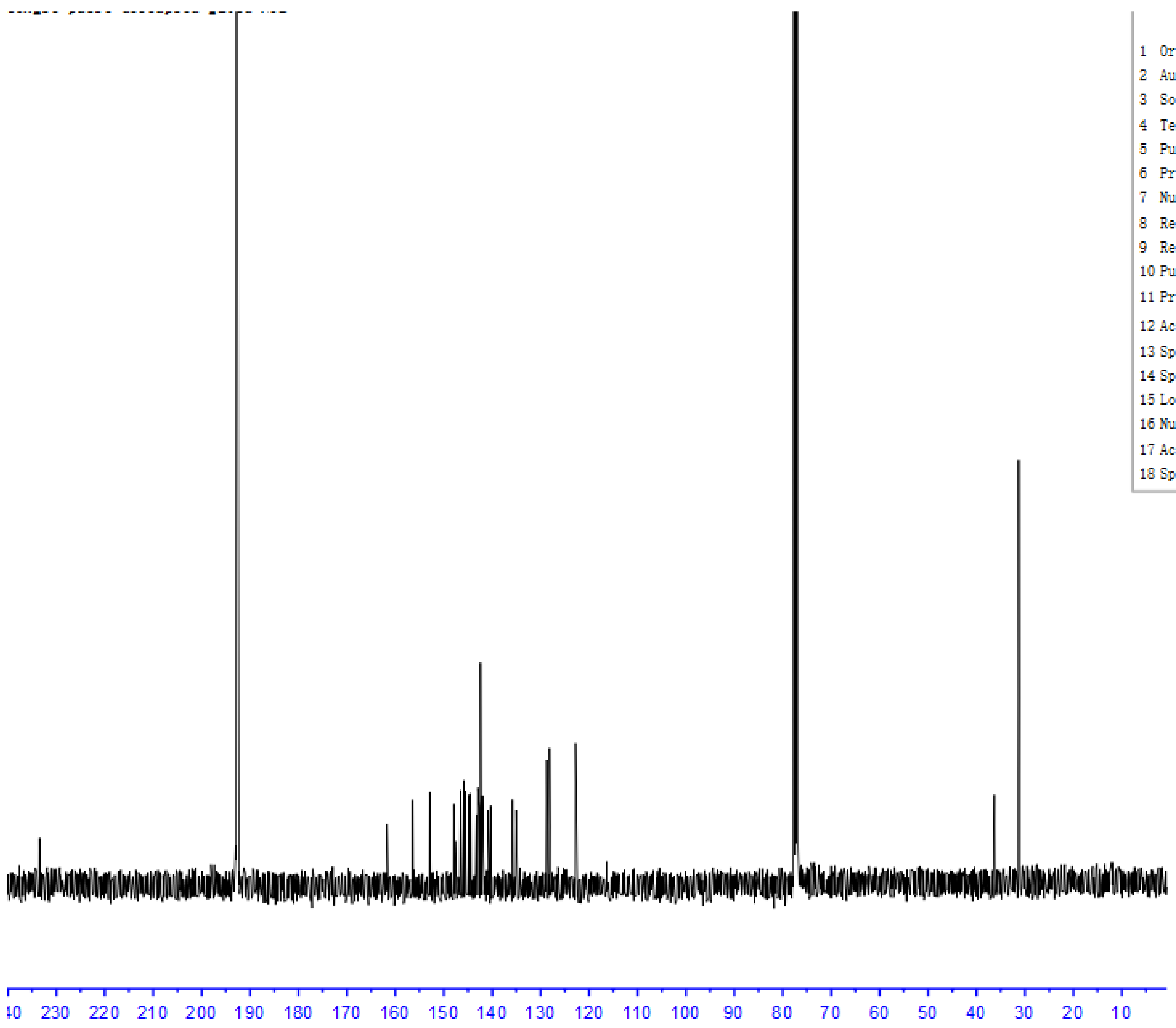


| | Parameter | Value |
|---|---|---|
| 1 | Origin | JEOL |
| 2 | Author | delta |
| 3 | Solvent | CHLOROFORM-D |
| 4 | Temperature | 21.7 |
| 5 | Pulse Sequence | carbon.jxp |
| 6 | Probe | 2803 |
| 7 | Number of Scans | 5120 |
| 8 | Receiver Gain | 50.0 |
| 9 | Relaxation Delay | 2.2891 |
| 10 | Pulse Width | 3.3767 |
| 11 | Presaturation Frequency | |
| 12 | Acquisition Time | 0.7109 |
| 13 | Spectrometer Frequency | 100.53 |
| 14 | Spectral Width | 30302.6 |
| 15 | Lowest Frequency | -72.5 |
| 16 | Nucleus | Carbon13 |
| 17 | Acquired Size | 26928 |
| 18 | Spectral Size | 52429 |

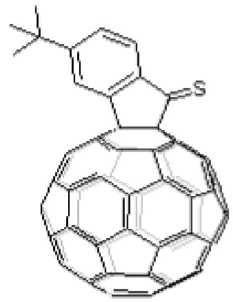


8a

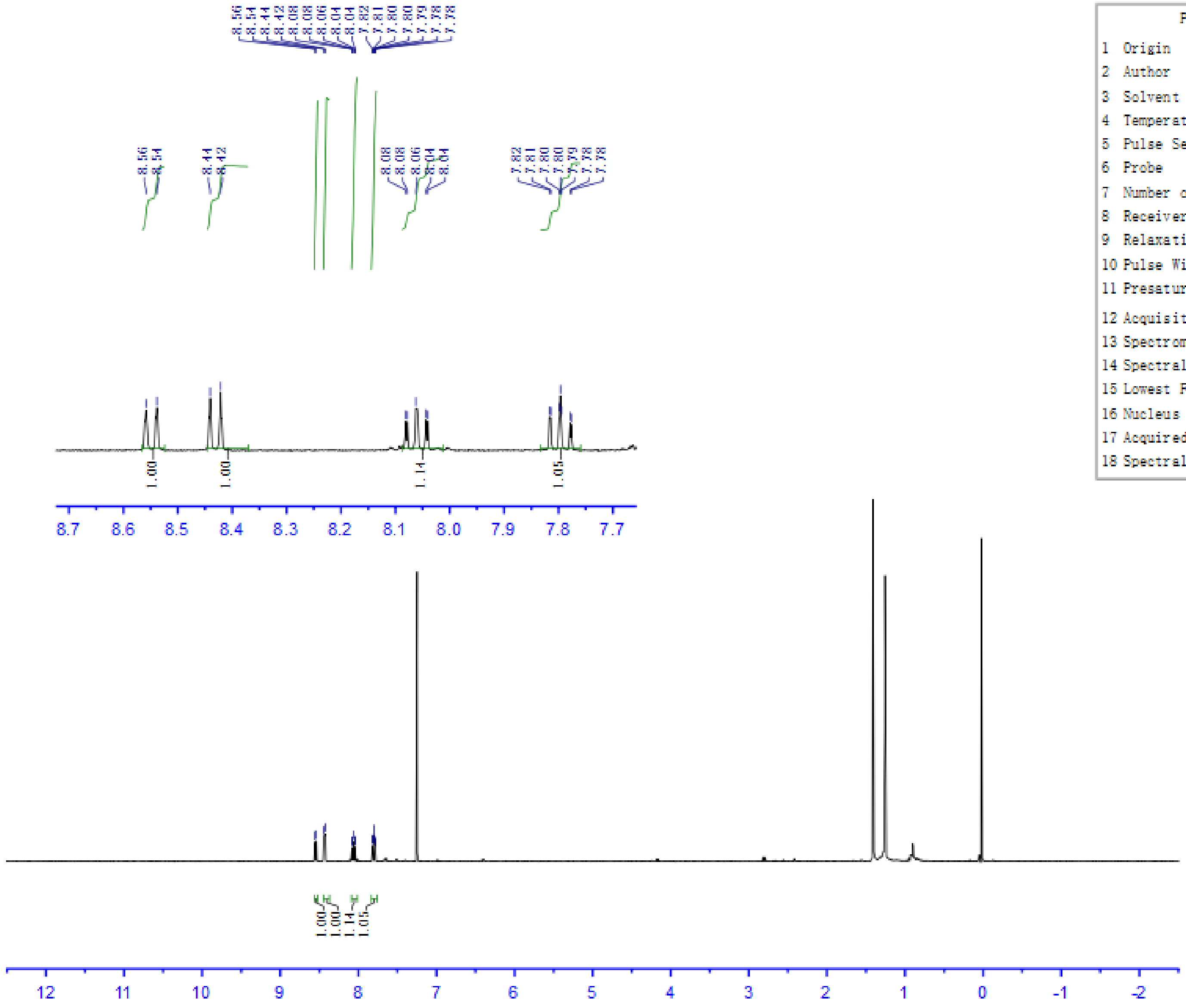


| Parameter | Value |
|---|---|
| 1 Origin | JEOL |
| 2 Author | delta |
| 3 Solvent | CHLOROFORM-D |
| 4 Temperature | 22.1 |
| 5 Pulse Sequence | proton_auto.jxp |
| 6 Probe | 2803 |
| 7 Number of Scans | 32 |
| 8 Receiver Gain | 66.0 |
| 9 Relaxation Delay | 5.0000 |
| 10 Pulse Width | 3.0750 |
| 11 Presaturation Frequency | |
| 12 Acquisition Time | 3.1831 |
| 13 Spectrometer Frequency | 399.78 |
| 14 Spectral Width | 6006.0 |
| 15 Lowest Frequency | -1004.1 |
| 16 Nucleus | Proton |
| 17 Acquired Size | 23897 |
| 18 Spectral Size | 52430 |

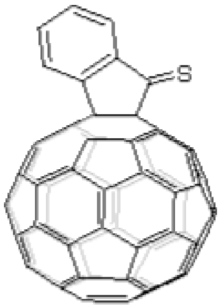


**5b**

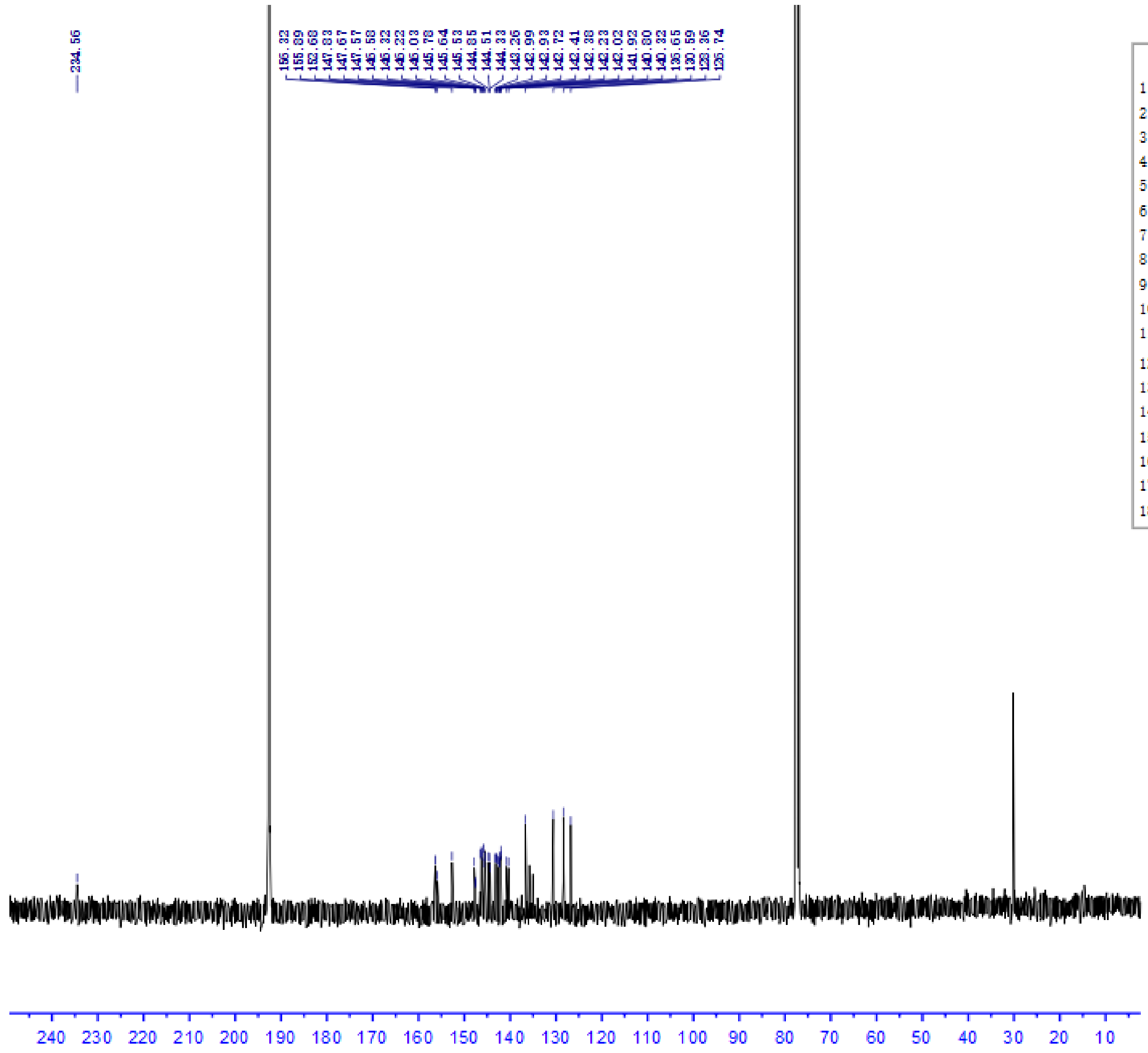


| | Parameter | Value |
|---|---|---|
| 1 | Origin | JEOL |
| 2 | Author | delta |
| 3 | Solvent | CHLOROFORM-D |
| 4 | Temperature | 22.1 |
| 5 | Pulse Sequence | carbon.jxp |
| 6 | Probe | 2803 |
| 7 | Number of Scans | 10240 |
| 8 | Receiver Gain | 50.0 |
| 9 | Relaxation Delay | 2.2891 |
| 10 | Pulse Width | 3.3767 |
| 11 | Presaturation Frequency | |
| 12 | Acquisition Time | 0.7109 |
| 13 | Spectrometer Frequency | 100.53 |
| 14 | Spectral Width | 30302.6 |
| 15 | Lowest Frequency | -72.5 |
| 16 | Nucleus | Carbon13 |
| 17 | Acquired Size | 26928 |
| 18 | Spectral Size | 52429 |

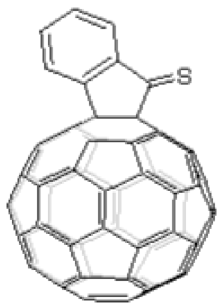


**5b**

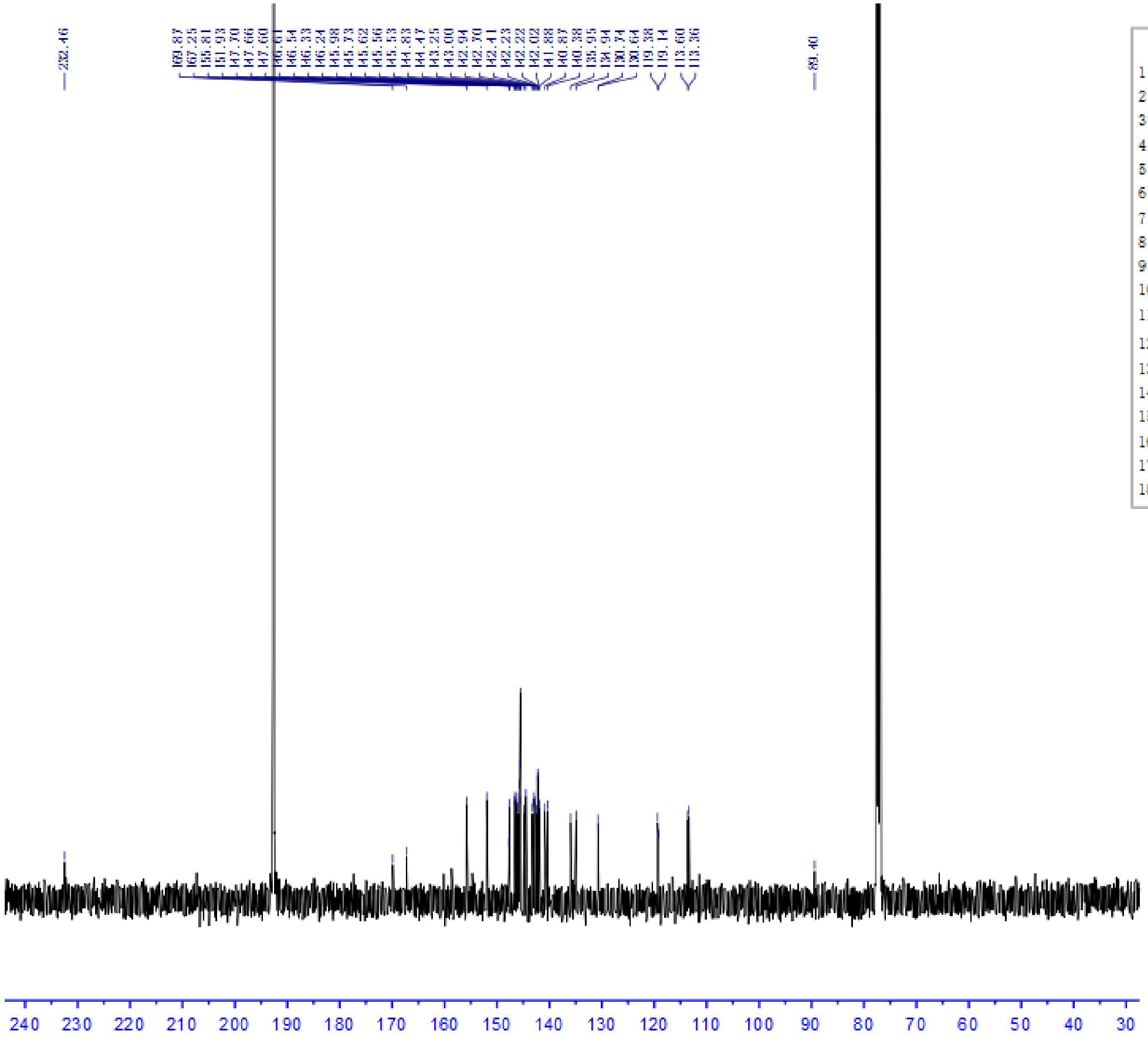


| Parameter | Value |
|---|---|
| 1 Origin | JEOL |
| 2 Author | delta |
| 3 Solvent | CHLOROFORM-D |
| 4 Temperature | 21.5 |
| 5 Pulse Sequence | carbon.jxp |
| 6 Probe | 2803 |
| 7 Number of Scans | 10240 |
| 8 Receiver Gain | 50.0 |
| 9 Relaxation Delay | 2.2891 |
| 10 Pulse Width | 3.3767 |
| 11 Presaturation Frequency | |
| 12 Acquisition Time | 0.7109 |
| 13 Spectrometer Frequency | 100.53 |
| 14 Spectral Width | 30302.6 |
| 15 Lowest Frequency | -72.5 |
| 16 Nucleus | Carbon13 |
| 17 Acquired Size | 26928 |
| 18 Spectral Size | 52429 |

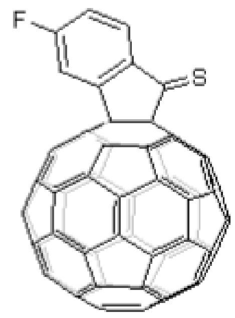


**5c**

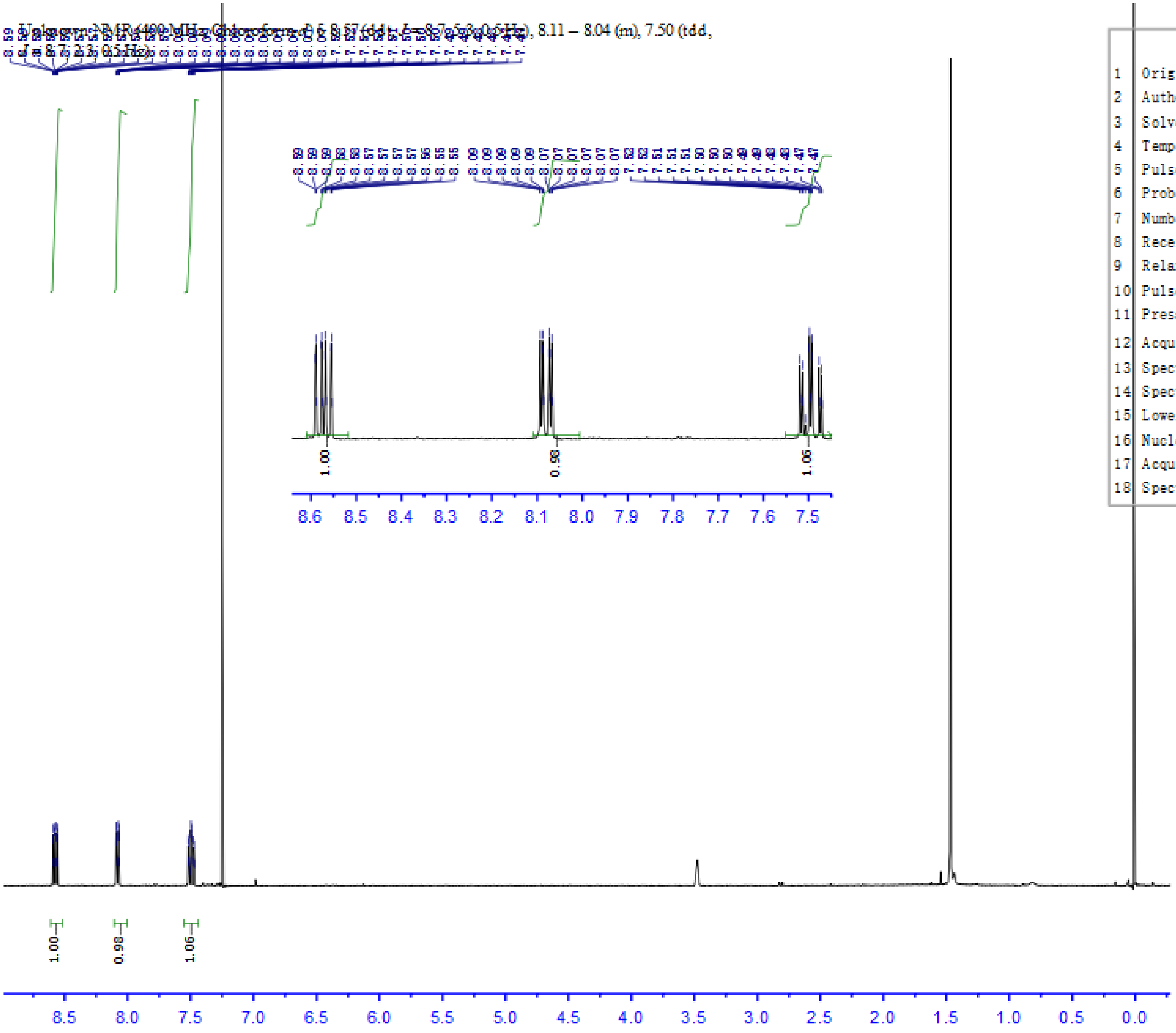


| | Parameter | Value |
|---|---|---|
| 1 | Origin | JEOL |
| 2 | Author | delta |
| 3 | Solvent | CHLOROFORM-D |
| 4 | Temperature | 21.8 |
| 5 | Pulse Sequence | proton_auto.jxp |
| 6 | Probe | 2803 |
| 7 | Number of Scans | 32 |
| 8 | Receiver Gain | 66.0 |
| 9 | Relaxation Delay | 5.0000 |
| 10 | Pulse Width | 3.0750 |
| 11 | Presaturation Frequency | |
| 12 | Acquisition Time | 3.1831 |
| 13 | Spectrometer Frequency | 399.78 |
| 14 | Spectral Width | 6006.0 |
| 15 | Lowest Frequency | -1004.1 |
| 16 | Nucleus | Proton |
| 17 | Acquired Size | 23897 |
| 18 | Spectral Size | 52430 |

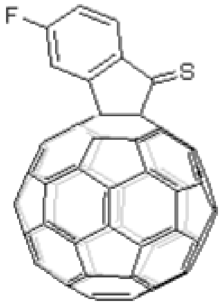

5c

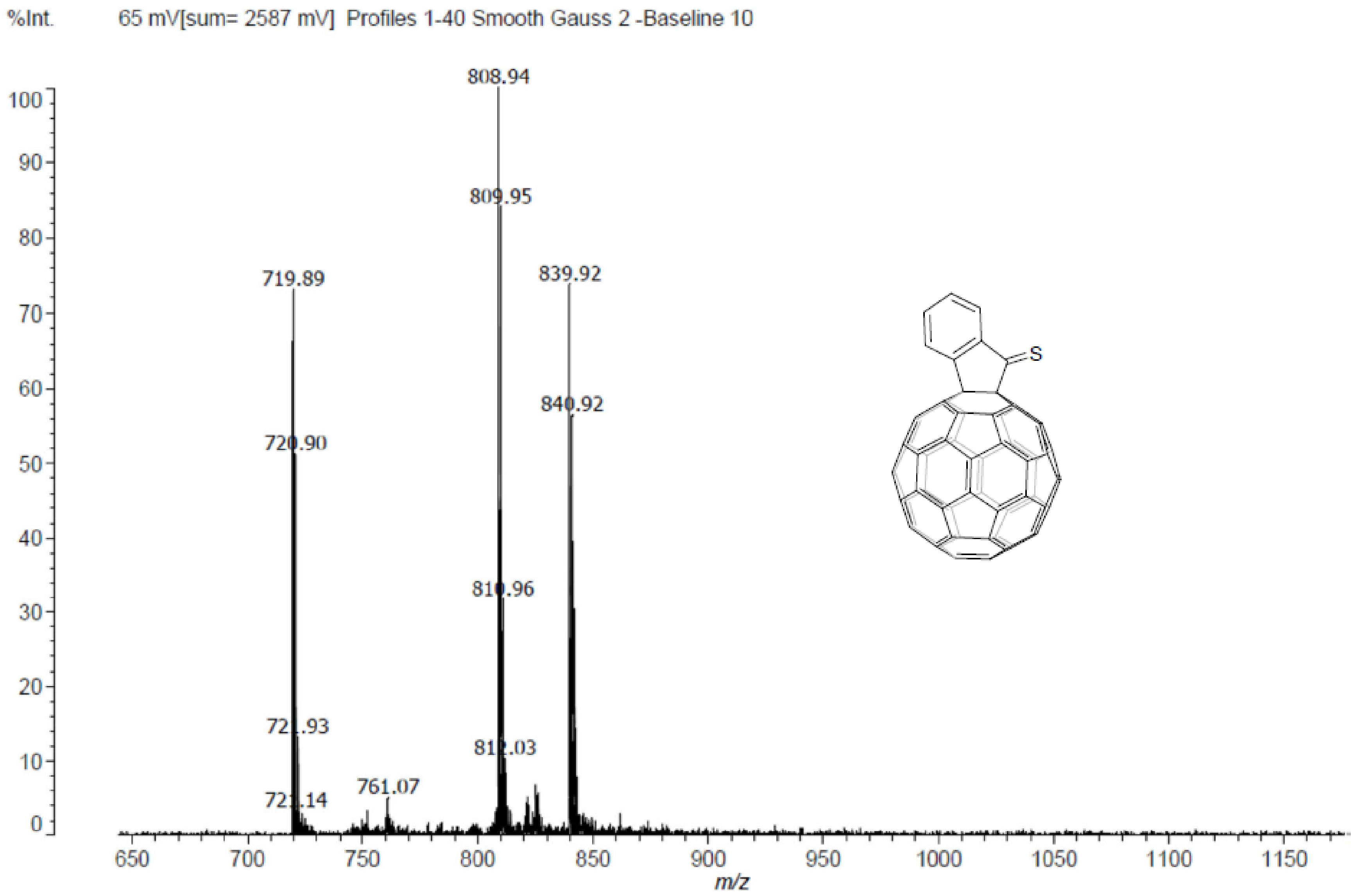
%Int.
65 mV[sum= 2587 mV] Profiles 1-40 Smooth Gauss 2 -Baseline 10
808.94
809.95
719.89
839.92
840.92
761.07
S
m/z

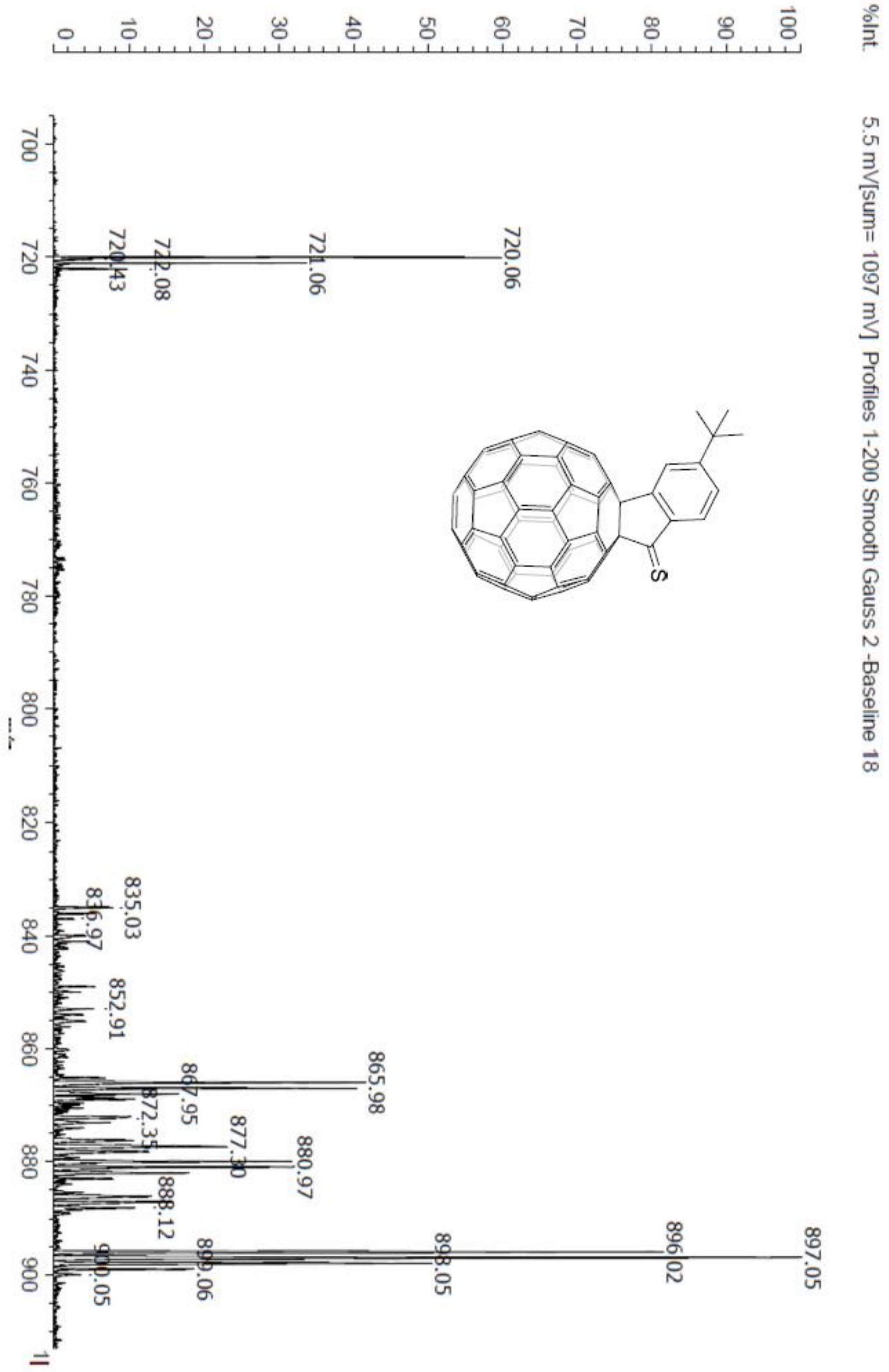

%Int.
5.5 mV[sum= 1097 mV] Profiles 1-200 Smooth Gauss 2 -Baseline 18
S
720.06
721.06
722.08
720.43
835.03
836.97
852.91
865.98
867.95
872.35
877.30
880.97
888.12
896.02
897.05
898.05
899.06
900.05

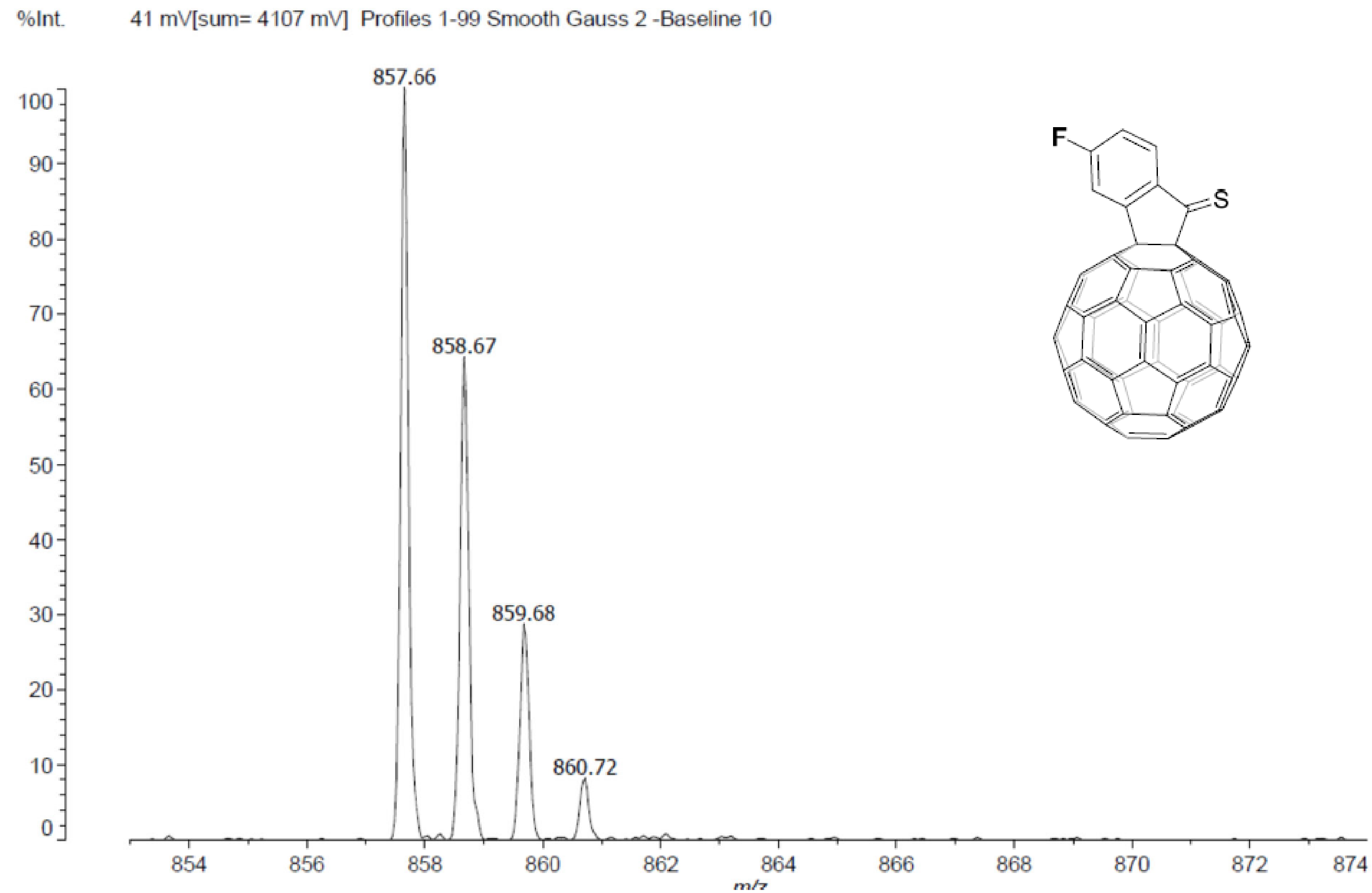
%Int.
41 mV[sum= 4107 mV]  Profiles 1-99 Smooth Gauss 2 -Baseline 10
857.66
858.67
859.68
860.72
0
10
20
30
40
50
60
70
80
90
100
854
856
858
860
862
864
866
868
870
872
874
m/z
F
S

## 8. Computational data

**Table S1.** Simulation for vibration of *t*-Bu-FIDO (left) and *t*-Bu-FIDS (right)

| *t*-Bu-FIDO vibration | | *t*-Bu-FIDS vibration | |
|---|---|---|---|
| Frequency ($cm^{-1}$) | Epsilon ($M^{-1}$ $cm^{-1}$) | Frequency ($cm^{-1}$) | Epsilon ($M^{-1}$ $cm^{-1}$) |
| 34.4656 | 0.20835 | 41.28579 | 0.00626 |
| 56.3838 | 20.62488 | 58.52742 | 29.00104 |
| 65.7884 | 3.01986 | 63.87735 | 24.46495 |
| 99.9959 | 63.89282 | 70.05813 | 14.2865 |
| 144.8204 | 11.71583 | 132.75737 | 28.53075 |
| 162.2506 | 22.83968 | 158.74273 | 5.73898 |
| 189.4856 | 5.06345 | 188.8313 | 0.52435 |
| 221.7946 | 1.29326 | 192.0298 | 36.5428 |
| 236.4787 | 21.73363 | 228.08132 | 3.27663 |
| 237.7582 | 0.74668 | 251.52542 | 1.61157 |
| 252.5937 | 0.01895 | 251.95696 | 0.21967 |
| 255.2707 | 0.23442 | 254.35761 | 0.06112 |
| 266.532 | 3.7195 | 262.38045 | 9.1858 |
| 269.2064 | 0.02519 | 269.3761 | 0.05319 |
| 273.5868 | 1.51068 | 274.9394 | 3.82632 |
| 288.5923 | 31.20964 | 286.39565 | 5.96694 |
| 313.9002 | 11.52845 | 304.0532 | 2.24385 |
| 333.7379 | 0.60964 | 323.09418 | 7.03449 |
| 336.0422 | 0.63751 | 336.39034 | 0.05494 |
| 337.6815 | 0.43121 | 338.53739 | 0.01082 |
| 338.6451 | 0.16021 | 342.54908 | 1.22797E-4 |
| 343.0459 | 0.01047 | 344.23011 | 0.24717 |
| 346.7 | 0.10126 | 346.88503 | 0.02133 |
| 353.506 | 3.67221 | 352.3317 | 5.37233 |
| 363.7724 | 6.47257 | 358.46175 | 22.91525 |
| 365.0149 | 0.05574 | 365.14683 | 2.937 |
| 375.3216 | 42.83918 | 365.33463 | 0.02175 |
| 394.7179 | 0.01314 | 392.84381 | 0.76831 |
| 398.865 | 9.74483 | 398.7671 | 7.24512 |
| 399.1133 | 0.07597 | 398.88119 | 0.24263 |
| 408.6214 | 0.03027 | 408.61395 | 0.04027 |
| 409.1693 | 0.00585 | 409.49255 | 11.16948 |
| 410.1316 | 19.52913 | 409.64008 | 5.31111E-4 |
| 432.509 | 0.51192 | 431.06157 | 0.94002 |

| | | | |
|---|---|---|---|
| 436.0605 | 2.56988 | 431.99684 | 0.77895 |
| 436.2287 | 0.29722 | 436.0308 | 1.00493 |
| 436.3337 | 0.14629 | 436.19932 | 0.06819 |
| 449.0373 | 9.95312 | 441.52508 | 2.40651 |
| 457.7539 | 1.83454 | 454.14473 | 5.3597 |
| 463.4829 | 18.33555 | 458.79326 | 2.7921 |
| 466.5332 | 1.54947 | 463.01867 | 18.71205 |
| 478.781 | 0.63076 | 477.49423 | 0.9309 |
| 484.5471 | 25.39546 | 483.22954 | 19.53456 |
| 490.9428 | 6.93553 | 491.08159 | 6.32465 |
| 491.9362 | 9.06327 | 491.95567 | 8.05106 |
| 516.5557 | 0.40623 | 508.84357 | 2.73087 |
| 529.0155 | 0.02112 | 522.36119 | 0.11093 |
| 531.8753 | 19.53838 | 524.05413 | 7.41189 |
| 535.8619 | 25.33697 | 533.30862 | 0.04924 |
| 536.2392 | 227.41176 | 536.15021 | 147.58175 |
| 537.1045 | 43.23532 | 536.57173 | 65.34471 |
| 537.3417 | 114.28852 | 536.75984 | 209.64682 |
| 539.05 | 116.81633 | 538.95917 | 118.11785 |
| 553.0171 | 67.59042 | 546.70616 | 0.52437 |
| 554.7822 | 1.48493 | 548.60761 | 73.43171 |
| 562.24 | 77.74727 | 561.69214 | 65.02186 |
| 566.0357 | 0.65969 | 563.85878 | 33.03154 |
| 566.6802 | 0.01126 | 566.24946 | 5.05945 |
| 568.2463 | 0.12637 | 566.45243 | 0.00655 |
| 570.475 | 30.86901 | 568.21119 | 0.11525 |
| 574.2364 | 1.07266 | 571.0099 | 17.75748 |
| 576.6103 | 1.57677 | 572.7756 | 0.67991 |
| 583.727 | 40.67671 | 576.93525 | 1.82331 |
| 584.2997 | 22.06562 | 583.69417 | 41.01059 |
| 591.0987 | 50.87286 | 585.66665 | 45.28047 |
| 596.7081 | 10.6222 | 591.22818 | 45.39996 |
| 604.025 | 88.94535 | 592.35014 | 2.87654 |
| 619.3688 | 1.56776 | 615.77004 | 34.09666 |
| 633.4068 | 16.05315 | 618.71206 | 1.36311 |
| 634.6143 | 0 | 620.13343 | 13.10797 |
| 662.0056 | 3.77302 | 652.48313 | 2.26757 |
| 675.3925 | 13.40367 | 672.72386 | 6.29867 |
| 679.7063 | 8.17182 | 677.54019 | 16.596 |
| 681.1937 | 0.02694 | 680.887 | 0.00596 |

| | | | |
|---|---|---|---|
| 687.895 | 8.20271 | 687.52862 | 10.21058 |
| 702.5326 | 100.65137 | 689.39241 | 5.50143 |
| 703.2299 | 26.9965 | 696.48417 | 61.067 |
| 712.7053 | 34.92978 | 709.18293 | 9.34816 |
| 714.7594 | 2.70254 | 713.37075 | 0.0312 |
| 717.7472 | 1.47515 | 717.5434 | 1.88587 |
| 719.4021 | 0.38153 | 718.82625 | 0.12911 |
| 721.7596 | 1.01758 | 721.28877 | 0.08192 |
| 722.1129 | 0.03591 | 721.96621 | 0.19473 |
| 724.0543 | 0.28761 | 723.79134 | 1.46113E-4 |
| 732.4877 | 2.61752 | 731.38967 | 0.0749 |
| 736.9883 | 0.7849 | 735.50808 | 5.65425 |
| 737.3125 | 0.00433 | 736.83549 | 0.38684 |
| 737.3806 | 17.02545 | 737.85305 | 0.26679 |
| 741.5694 | 26.27753 | 739.66199 | 5.66448 |
| 742.0347 | 0.02258 | 740.59789 | 20.6912 |
| 743.121 | 18.09056 | 742.44542 | 0.00178 |
| 743.461 | 1.1553 | 744.04772 | 2.7942 |
| 746.8782 | 0.98549 | 746.42849 | 6.59843 |
| 751.4609 | 0.03238 | 749.68531 | 6.64097 |
| 752.0669 | 0.72036 | 751.47457 | 0.08557 |
| 753.2589 | 4.55419 | 752.91723 | 0.17868 |
| 754.2125 | 4.90071 | 753.69742 | 0.17463 |
| 754.4455 | 0.36856 | 754.11866 | 0.69648 |
| 754.6922 | 0.32615 | 754.19521 | 0.01152 |
| 756.1601 | 0.26801 | 756.69209 | 0.16388 |
| 761.121 | 49.65669 | 757.80127 | 20.78293 |
| 763.5864 | 0.98013 | 763.15219 | 2.02657 |
| 767.5159 | 0.00104 | 767.57334 | 2.60685 |
| 767.8726 | 1.76955 | 767.7029 | 0.05099 |
| 772.0587 | 2.86057 | 771.10901 | 0.32745 |
| 773.3048 | 6.49505 | 771.47546 | 0.61095 |
| 785.0952 | 1.03 | 785.02251 | 0.87989 |
| 792.0691 | 9.34002 | 791.62032 | 11.12073 |
| 798.1055 | 1.15017 | 797.7365 | 0.10413 |
| 804.0706 | 0.22376 | 803.82974 | 0.10327 |
| 805.2351 | 0.8249 | 805.11066 | 0.06613 |
| 815.2576 | 18.26762 | 815.46969 | 0.71979 |
| 823.1936 | 1.43255 | 816.19809 | 7.88643 |
| 838.3503 | 27.71092 | 826.42431 | 84.66891 |

| | | | |
|---|---|---|---|
| 841.9506 | 4.57718 | 840.42441 | 51.28321 |
| 847.9782 | 9.8392 | 841.46528 | 1.92907 |
| 848.0155 | 28.07252 | 843.79723 | 1.83559 |
| 867.7 | 93.44394 | 846.66486 | 39.08205 |
| 868.9886 | 33.70144 | 865.08163 | 69.30077 |
| 912.5097 | 7.573 | 913.21227 | 7.79143 |
| 939.1106 | 7.93707 | 936.76408 | 9.28281 |
| 948.824 | 8.29604 | 943.34956 | 5.29226 |
| 950.299 | 18.41766 | 946.81901 | 1.86084 |
| 959.2558 | 0.29278 | 954.25024 | 0.1182 |
| 962.5802 | 90.38123 | 955.1276 | 21.3777 |
| 965.2645 | 0.00992 | 964.81967 | 0.11082 |
| 968.7203 | 95.69082 | 968.52894 | 16.92122 |
| 973.907 | 0.35023 | 970.70561 | 0.41589 |
| 982.9752 | 56.62287 | 983.10566 | 20.51405 |
| 986.2878 | 2.25501 | 986.18243 | 3.42829 |
| 986.9184 | 0.14067 | 989.95564 | 0.13441 |
| 989.7071 | 0.12617 | 991.26591 | 0.02474 |
| 991.8686 | 4.0221E-4 | 996.68687 | 2.0753E-4 |
| 1025.5851 | 233.74062 | 1018.23772 | 11.29818 |
| 1055.348 | 24.45847 | 1050.63694 | 22.93456 |
| 1060.5345 | 0.0158 | 1055.58632 | 0.02235 |
| 1062.897 | 3.04883 | 1056.09692 | 3.80002 |
| 1080.6198 | 0.99973 | 1078.20953 | 0.26896 |
| 1093.2175 | 0.48681 | 1086.41534 | 169.46892 |
| 1100.8299 | 5.84115 | 1089.2816 | 0.02322 |
| 1104.7458 | 90.15838 | 1101.6556 | 2.50371 |
| 1105.8245 | 0.13493 | 1105.3423 | 5.84236E-4 |
| 1113.9864 | 1.22799 | 1114.10584 | 0.37644 |
| 1122.8568 | 18.2349 | 1121.95498 | 37.1422 |
| 1125.2728 | 27.94099 | 1124.22693 | 15.94856 |
| 1131.4563 | 0.14104 | 1129.23097 | 103.57075 |
| 1143.2387 | 11.53683 | 1131.71816 | 0.11944 |
| 1160.4013 | 170.99451 | 1144.31035 | 71.04546 |
| 1168.6398 | 3.49018 | 1168.00616 | 4.10097 |
| 1172.6174 | 0.01973 | 1169.553 | 0.01456 |
| 1186.9139 | 2.12828 | 1183.04375 | 47.96023 |
| 1202.5067 | 0.771 | 1188.53237 | 31.39894 |
| 1207.4678 | 43.53333 | 1202.82529 | 0.70654 |
| 1211.5489 | 25.39216 | 1211.4575 | 11.74819 |

| | | | |
|---|---|---|---|
| 1218.8862 | 32.04286 | 1213.91615 | 83.68797 |
| 1220.1317 | 5.86215 | 1219.1053 | 43.24274 |
| 1236.6499 | 9.30177 | 1220.5232 | 5.10268 |
| 1239.9628 | 3.01112 | 1229.57257 | 9.20185 |
| 1245.3991 | 0.42668 | 1233.62481 | 6.30956 |
| 1246.4254 | 4.66754 | 1245.4504 | 0.96634 |
| 1248.44 | 8.7624 | 1246.41231 | 3.80799 |
| 1255.2237 | 95.62169 | 1248.43749 | 14.36907 |
| 1259.6281 | 4.13943 | 1254.57413 | 20.61309 |
| 1263.8601 | 6.31304E-4 | 1259.32182 | 3.85244 |
| 1266.6712 | 474.86908 | 1263.472 | 0.01439 |
| 1274.7113 | 21.03655 | 1274.49411 | 3.16 |
| 1283.4757 | 8.97266 | 1283.08415 | 3.92479 |
| 1296.7947 | 17.54598 | 1291.83885 | 155.77012 |
| 1298.191 | 3.0795 | 1296.72811 | 11.20417 |
| 1303.1737 | 22.87613 | 1298.13628 | 3.06741 |
| 1303.8642 | 0.01622 | 1303.6513 | 0.00973 |
| 1309.4025 | 61.74688 | 1307.34485 | 58.30849 |
| 1315.3392 | 0.00364 | 1312.6777 | 1.29569 |
| 1318.5228 | 82.85791 | 1315.28882 | 5.69789E-4 |
| 1333.7322 | 2.33968 | 1333.48261 | 4.03485 |
| 1333.7483 | 0.34398 | 1333.49493 | 0.3242 |
| 1335.838 | 3.8767 | 1335.19114 | 66.16436 |
| 1337.8745 | 0.00567 | 1337.90325 | 0.01349 |
| 1342.7421 | 0.68097 | 1341.86135 | 120.05132 |
| 1343.5121 | 1.00543 | 1343.27221 | 0.69388 |
| 1347.4341 | 13.76535 | 1345.90846 | 307.21881 |
| 1367.434 | 1.60721 | 1348.32225 | 165.46625 |
| 1368.2717 | 0.03382 | 1366.15354 | 6.6771 |
| 1372.5239 | 14.67346 | 1368.35367 | 0.02873 |
| 1373.1734 | 0.18913 | 1369.17737 | 0.33446 |
| 1373.5059 | 0.75634 | 1373.16945 | 0.09429 |
| 1379.0043 | 0.06972 | 1373.69794 | 0.9426 |
| 1381.0723 | 1.72191 | 1379.36664 | 0.13854 |
| 1386.4264 | 8.01893 | 1381.29118 | 3.24336 |
| 1388.6081 | 0.02385 | 1387.11984 | 0.5905 |
| 1426.6652 | 14.46755 | 1388.74846 | 0.00247 |
| 1427.8279 | 12.6226 | 1415.12424 | 9.34811 |
| 1436.7483 | 0.08413 | 1415.6553 | 12.38831 |
| 1451.3532 | 5.02168 | 1436.49387 | 0.03444 |

| | | | |
|---|---|---|---|
| 1454.6627 | 12.6149 | 1448.14378 | 6.34423 |
| 1455.3865 | 1.39606 | 1451.37584 | 5.94539 |
| 1455.9282 | 62.40207 | 1454.64825 | 11.88606 |
| 1457.3597 | 76.94078 | 1455.44523 | 0.4632 |
| 1458.7668 | 26.70124 | 1456.0986 | 38.46669 |
| 1458.9789 | 13.09766 | 1457.86654 | 72.50502 |
| 1460.6141 | 15.64251 | 1459.11353 | 25.75798 |
| 1461.2177 | 24.70082 | 1459.53921 | 35.72259 |
| 1464.7273 | 0.1528 | 1462.19523 | 16.94767 |
| 1468.0678 | 1.02556 | 1465.38925 | 0.82301 |
| 1476.2893 | 11.72507 | 1468.04619 | 1.05406 |
| 1496.4393 | 20.94299 | 1476.47797 | 7.38818 |
| 1512.9674 | 0 | 1496.92738 | 21.94074 |
| 1516.3255 | 0.30756 | 1497.60019 | 9.54358E-4 |
| 1518.3553 | 2.1524 | 1501.32198 | 0.26821 |
| 1526.6318 | 3.22495 | 1503.90582 | 0.68072 |
| 1529.7162 | 10.83753 | 1516.92132 | 46.87178 |
| 1537.9635 | 7.869 | 1523.37056 | 16.53529 |
| 1538.8749 | 17.61181 | 1525.19024 | 10.58319 |
| 1539.9691 | 0.05129 | 1527.84125 | 1.94138 |
| 1540.0296 | 0.05854 | 1536.61572 | 25.86335 |
| 1550.6765 | 34.6692 | 1539.87697 | 0.16849 |
| 1551.3752 | 1.08698 | 1540.18518 | 0.01695 |
| 1559.7021 | 2.23372 | 1551.4227 | 0.96224 |
| 1571.1207 | 1.04412 | 1559.93265 | 2.02574 |
| 1600.1226 | 1.79335 | 1571.5715 | 1.04617 |
| 1602.7298 | 4.58646 | 1601.56017 | 1.18594 |
| 1604.1443 | 0.00124 | 1602.95591 | 9.51132 |
| 1605.7697 | 0.14062 | 1604.22586 | 0.02599 |
| 1608.4171 | 0.94054 | 1605.93331 | 0.25764 |
| 1609.5966 | 0.05701 | 1608.47635 | 1.15701 |
| 1613.3207 | 5.95694 | 1609.45965 | 0.05502 |
| 1614.6035 | 0.27821 | 1611.82438 | 46.9399 |
| 1615.965 | 0.67026 | 1614.67171 | 0.20164 |
| 1628.1804 | 4.63973 | 1615.07738 | 12.37073 |
| 1629.8741 | 0.08812 | 1617.16958 | 16.66188 |
| 1634.2198 | 121.37195 | 1629.38957 | 1.4689 |
| 1654.8836 | 418.24265 | 1630.36492 | 5.42714E-4 |
| 1814.8508 | 614.91736 | 1649.81408 | 453.02441 |
| 3046.1957 | 32.5758 | 3031.97582 | 31.32994 |

| | | | |
|---|---|---|---|
| 3047.1271 | 30.0478 | 3032.89263 | 29.11754 |
| 3053.974 | 33.02894 | 3039.3623 | 35.87596 |
| 3113.3712 | 2.09761 | 3103.02384 | 1.84308 |
| 3113.7136 | 2.04754 | 3103.26496 | 1.67925 |
| 3117.5306 | 28.00843 | 3106.85484 | 27.34062 |
| 3121.3846 | 138.81686 | 3110.55311 | 136.00149 |
| 3124.0959 | 16.55779 | 3113.68148 | 15.05937 |
| 3126.4785 | 46.05364 | 3116.05243 | 43.87036 |
| 3200.5443 | 9.90985 | 3192.95871 | 12.39887 |
| 3216.3872 | 27.91473 | 3209.0889 | 16.86476 |
| 3229.2964 | 1.92496 | 3220.4556 | 2.30941 |

**Table S2.** Cartesian coordinates of optimized structures

*t*-Bu-FIDS

| | | | |
|---|---|---|---|
| C | 1.00598 | 2.1391 | 1.17689 |
| C | -0.03928 | 3.01582 | 0.72509 |
| C | -1.24547 | 3.12311 | 1.42498 |
| C | -1.45971 | 2.30445 | 2.59331 |
| C | -0.4555 | 1.42043 | 3.00295 |
| C | 0.79638 | 1.34402 | 2.28151 |
| C | 1.00597 | 2.13912 | -1.17688 |
| C | -0.03928 | 3.01583 | -0.72506 |
| C | -1.24548 | 3.12313 | -1.42494 |
| C | -2.50113 | 3.21691 | 0.69702 |
| C | -2.8478 | 1.88073 | 2.59866 |
| C | -3.18407 | 0.59466 | 3.02687 |
| C | -2.1433 | -0.31997 | 3.46816 |
| C | -0.80864 | 0.08469 | 3.44868 |
| C | 0.22594 | -0.83133 | 3.00326 |
| C | 1.22464 | -0.07303 | 2.28198 |
| C | 1.84298 | -0.61701 | 1.1783 |
| C | 0.79636 | 1.34405 | -2.28151 |
| C | 1.22462 | -0.073 | -2.282 |
| C | 1.84297 | -0.617 | -1.17833 |
| C | 1.45793 | -1.92571 | -0.72598 |
| C | 1.45793 | -1.92572 | 0.72593 |
| C | 0.51337 | -2.68585 | 1.42538 |
| C | -0.11868 | -2.12444 | 2.5939 |

| | | | |
|---|---|---|---|
| C | -1.50871 | -2.54327 | 2.59956 |
| C | -2.50137 | -1.65884 | 3.02641 |
| C | -3.49371 | 2.44585 | 1.42455 |
| C | -4.60927 | -1.79648 | 0. |
| C | -5.01333 | -0.46053 | 0.00001 |
| C | -4.79646 | 0.366 | -1.17545 |
| C | -4.17695 | -0.17553 | -2.30386 |
| C | -3.75495 | -1.56892 | -2.30373 |
| C | -2.93816 | -3.2815 | -0.7271 |
| C | -2.93815 | -3.2815 | 0.72707 |
| C | -3.96996 | -2.36338 | 1.17541 |
| C | -3.75493 | -1.56895 | 2.30373 |
| C | -4.17693 | -0.17556 | 2.30388 |
| C | -4.79645 | 0.36598 | 1.17548 |
| C | -4.44809 | 1.7025 | -0.72698 |
| C | -3.49372 | 2.44586 | -1.4245 |
| C | -2.84782 | 1.88077 | -2.59862 |
| C | -3.1841 | 0.5947 | -3.02684 |
| C | -2.14333 | -0.31993 | -3.46816 |
| C | -2.5014 | -1.6588 | -3.02642 |
| C | -1.50873 | -2.54324 | -2.59959 |
| C | -1.73227 | -3.37127 | -1.42536 |
| C | -1.73226 | -3.37129 | 1.42532 |
| C | -0.47868 | -3.46078 | 0.69705 |
| C | -0.47868 | -3.46077 | -0.6971 |
| C | 0.51336 | -2.68583 | -1.42543 |
| C | -0.1187 | -2.12441 | -2.59393 |
| C | 0.22592 | -0.83129 | -3.00328 |
| C | -0.80866 | 0.08473 | -3.44868 |
| C | -0.45552 | 1.42046 | -3.00294 |
| C | -1.45973 | 2.30448 | -2.59328 |
| C | -4.44808 | 1.70249 | 0.72703 |
| C | -2.50114 | 3.21692 | -0.69697 |
| C | -3.96997 | -2.36336 | -1.17542 |
| C | 1.91315 | 1.75312 | 0. |
| C | 3.23846 | 2.57724 | 0. |
| C | 2.38106 | 0.2137 | -0.00001 |
| C | 4.34594 | 1.64316 | -0.00002 |
| C | 3.90512 | 0.31702 | -0.00003 |
| C | 5.72291 | 1.92241 | -0.00003 |
| C | 4.814 | -0.73938 | -0.00003 |

| | | | |
|---|---|---|---|
| C | 6.61988 | 0.86839 | -0.00002 |
| H | 6.06049 | 2.95436 | -0.00004 |
| C | 6.19186 | -0.48229 | -0.00002 |
| H | 4.44511 | -1.75829 | -0.00001 |
| H | 7.6827 | 1.08941 | -0.00003 |
| S | 3.32203 | 4.21586 | 0.00002 |
| C | 7.24049 | -1.6069 | 0.00001 |
| C | 8.12577 | -1.47908 | 1.26382 |
| C | 6.60141 | -3.00847 | 0.00002 |
| C | 8.1258 | -1.47911 | -1.26379 |
| H | 8.65073 | -0.51918 | 1.30301 |
| H | 7.52506 | -1.57071 | 2.1757 |
| H | 8.88213 | -2.27269 | 1.27413 |
| H | 5.98217 | -3.17634 | -0.88837 |
| H | 7.38906 | -3.76967 | 0.00003 |
| H | 5.98215 | -3.17633 | 0.88839 |
| H | 8.88216 | -2.27274 | -1.27405 |
| H | 7.52511 | -1.57077 | -2.17567 |
| H | 8.65077 | -0.51922 | -1.30299 |

*t*-Bu-FIDO

| | | | |
|---|---|---|---|
| C | 0.99156 | 2.24653 | 1.17583 |
| C | -0.07749 | 3.0951 | 0.72516 |
| C | -1.28687 | 3.1639 | 1.42562 |
| C | -1.47522 | 2.33851 | 2.59391 |
| C | -0.44394 | 1.48588 | 3.00429 |
| C | 0.80936 | 1.44812 | 2.28348 |
| C | 0.99156 | 2.24652 | -1.17584 |
| C | -0.07749 | 3.09509 | -0.72518 |
| C | -1.28687 | 3.16389 | -1.42564 |
| C | -2.54451 | 3.21796 | 0.69714 |
| C | -2.84925 | 1.87091 | 2.59862 |
| C | -3.14523 | 0.57504 | 3.0269 |
| C | -2.07641 | -0.30677 | 3.46815 |
| C | -0.75508 | 0.13926 | 3.44932 |
| C | 0.30755 | -0.74374 | 3.0031 |
| C | 1.28213 | 0.04541 | 2.28162 |
| C | 1.92007 | -0.47928 | 1.1788 |
| C | 0.80936 | 1.44811 | -2.28349 |
| C | 1.28212 | 0.0454 | -2.28163 |

| | | | |
|---|---|---|---|
| C | 1.92006 | -0.47929 | -1.1788 |
| C | 1.57421 | -1.7992 | -0.72602 |
| C | 1.57421 | -1.7992 | 0.72603 |
| C | 0.65337 | -2.58817 | 1.42522 |
| C | 0.00374 | -2.04684 | 2.59365 |
| C | -1.37244 | -2.50902 | 2.59943 |
| C | -2.39238 | -1.65614 | 3.02631 |
| C | -3.51252 | 2.4156 | 1.42463 |
| C | -4.49504 | -1.85983 | 0.00001 |
| C | -4.94048 | -0.53711 | 0.00001 |
| C | -4.74945 | 0.29581 | -1.17545 |
| C | -4.11343 | -0.22604 | -2.30385 |
| C | -3.64819 | -1.60561 | -2.30374 |
| C | -2.77819 | -3.29176 | -0.72709 |
| C | -2.77819 | -3.29176 | 0.72711 |
| C | -3.83825 | -2.40636 | 1.17545 |
| C | -3.64819 | -1.6056 | 2.30375 |
| C | -4.11343 | -0.22603 | 2.30386 |
| C | -4.74945 | 0.29581 | 1.17546 |
| C | -4.44265 | 1.64248 | -0.72704 |
| C | -3.51252 | 2.4156 | -1.42464 |
| C | -2.84926 | 1.8709 | -2.59863 |
| C | -3.14524 | 0.57502 | -3.0269 |
| C | -2.07641 | -0.30678 | -3.46815 |
| C | -2.39239 | -1.65615 | -3.02631 |
| C | -1.37245 | -2.50903 | -2.59942 |
| C | -1.56996 | -3.34365 | -1.4252 |
| C | -1.56995 | -3.34364 | 1.42521 |
| C | -0.31413 | -3.39387 | 0.69701 |
| C | -0.31414 | -3.39387 | -0.697 |
| C | 0.65337 | -2.58817 | -1.42521 |
| C | 0.00374 | -2.04685 | -2.59365 |
| C | 0.30754 | -0.74376 | -3.0031 |
| C | -0.75509 | 0.13925 | -3.44932 |
| C | -0.44395 | 1.48586 | -3.00429 |
| C | -1.47523 | 2.33849 | -2.59392 |
| C | -4.44265 | 1.64248 | 0.72704 |
| C | -2.54451 | 3.21796 | -0.69715 |
| C | -3.83825 | -2.40636 | -1.17544 |
| C | 1.89602 | 1.8776 | -0.00001 |
| C | 3.19726 | 2.76483 | 0.00001 |

| | | | |
|---|---|---|---|
| C | 2.43033 | 0.36846 | -0.00001 |
| C | 4.35492 | 1.86332 | 0.00001 |
| C | 3.95696 | 0.53105 | -0.00001 |
| C | 5.71146 | 2.20723 | 0. |
| C | 4.90787 | -0.48977 | -0.00001 |
| C | 6.65175 | 1.18823 | -0.00002 |
| H | 6.00494 | 3.25293 | -0.00002 |
| C | 6.2745 | -0.17643 | -0.00001 |
| H | 4.58119 | -1.52307 | 0.00002 |
| H | 7.70552 | 1.44922 | -0.00005 |
| C | 7.36702 | -1.26026 | 0. |
| C | 8.24658 | -1.09905 | 1.2637 |
| C | 6.78296 | -2.68569 | 0.00005 |
| C | 8.24654 | -1.09908 | -1.26373 |
| H | 8.73441 | -0.11971 | 1.30309 |
| H | 7.64959 | -1.21343 | 2.17548 |
| H | 9.0329 | -1.86303 | 1.27457 |
| H | 6.1707 | -2.87771 | -0.88829 |
| H | 7.5997 | -3.41564 | 0.00007 |
| H | 6.1707 | -2.87765 | 0.88841 |
| H | 9.03283 | -1.8631 | -1.27464 |
| H | 7.64951 | -1.21344 | -2.17549 |
| H | 8.7344 | -0.11977 | -1.30313 |
| O | 3.19067 | 3.97694 | 0.00001 |

# Reference

[1] Shui, Q.-J.; Shan, S.; Zhai, Y.-C.; Aoyagi, S.; Izawa, S.; Huda, M.; Yu, C.-Y.; Zuo, L.; Chen, H.; Lin, H.-S.; Matsuo, Y. *J. Am. Chem. Soc*. **2023**, *145*, 27307–27315.

[2] Lin, H.-S.; Ma, Y.; Xiang, R.; Manzhos, S.; Jeon, I.; Maruyama, S.; Matsuo, Y. *Commun. Chem*. **2021**, *4*, 74.

[3] Becke, A. D. *J. Chem. Phys*. **1993**, *98*, 5648–5652.

[4] Lin, H.-S.; Jeon, I.; Chen, Y.; Yang, X.-Y.; Nakagawa, T.; Maruyama, S; Matsuo, Y. *Chem.Mater*. **2019**, *31* ,8432–8439.

[5] Matsuo, Y.; Yu, Y.; Yang, X. Y.; Ueno, H.; Okada, H.; Shibuya, H.; Choi, Y. S.; Jin, Y. W. *J. Org. Chem.* **2019**, *84*, 6270–6277.

[6] Frisch, M. J.; Trucks, G. W.; Schlegel, H. B.; Scuseria, G. E.; Robb, M. A.; Cheeseman, J. R.; Scalmani, G.; Barone, V.; Petersson, G. A.; Nakatsuji, H.; Li, X.; Caricato, M.; Marenich, A. V.; Bloino, J.; Janesko, B. G.; Gomperts, R.; Mennucci, B.; Hratchian, H. P.; Ortiz, J. V.; Izmaylov, A. F.; Sonnenberg, J. L.; Williams-Young, D.; Ding, F.; Lipparini, F.; Egidi, F.; Goings, J.; Peng, B.; Petrone,

A.; Henderson, T.; Ranasinghe, D.; Zakrzewski, V. G.; Gao, J.; Rega, N.; Zheng, G.; Liang, W.; Hada, M.; Ehara, M.; Toyota, K.; Fukuda, R.; Hasegawa, J.; Ishida, M.; Nakajima, T.; Honda, Y.; Kitao, O.; Nakai, H.; Vreven, T.; Throssell, K.; Montgomery, J., J. A.; Peralta, J. E.; Ogliaro, F.; Bearpark, M.; Heyd, J. J.; Brothers, E. N.; Kudin, K. N.; Staroverov, V. N.; Keith, T. A.; Kobayashi, R.; Normand, J.; Raghavachari, K.; Rendell, A. P.; Burant, J. C.; Iyengar, S. S.;Tomasi, J.; Cossi, M.; Millam, J. M.; Klene, M.; Adamo, C.; Cammi, R.; Ochterski, J. W.; Martin, R. L.; Morokuma, K.; Farkas, O.; Foresman, J. B.; Fox, D. J. Gaussian 16, Revision C.01; Gaussian, Inc.: Wallingford CT, 2019.
